# Individual factors associated with arterial and limb occlusion pressure in blood flow restriction: a scoping review of predictors and measurement methods

**DOI:** 10.3389/fphys.2026.1958680

**Published:** 2026-09-15

**Authors:** Robert Trybulski, Ireneusz Ryszkiel, Aneta Teległów, Zofia Ignasiak, Agata Stanek

**Affiliations:** 1 Faculty of Medicine, Katowice Business University, Katowice, Poland; 2 Provita Żory Medical Center, Żory, Poland; 3 Department of Internal Medicine, Metabolic Disease and Angiology, Faculty of Health Sciences in Katowice, Medical University of Silesia, Katowice, Poland; 4 Department of Health Promotion, Institute of Basic Sciences, Faculty of Motor Rehabilitation, University of Physical Culture in Kraków, Kraków, Poland; 5 Department of Preclinical Sciences, Pharmacology and Medical Diagnostics, Faculty of Medicine, Wrocław University of Science and Technology, Wrocław, Poland

**Keywords:** anthropometry, individualized, ischemia, KAATSU, morphology, vascular

## Abstract

**Objectives:**

This scoping review examined which individual characteristics are associated with arterial or limb occlusion pressure (AOP/LOP) and summarized the methodological and analytical approaches used to study these relationships in blood flow restriction (BFR) research. Design: Scoping review.

**Methods:**

The review followed PRISMA-ScR guidance. Searches were conducted on July 30, 2025, in PubMed, Scopus, and Web of Science, together with grey-literature searches, citation tracking, and expert consultation. Primary human studies were eligible when they quantitatively examined at least one individual characteristic as a predictor, correlate, stratification factor, or effect modifier of AOP/LOP, the pressure required to achieve occlusion, or an individualized response to BFR. Studies were excluded when participant characteristics were reported descriptively only or when BFR efficacy or safety was assessed without examining its relationship with an eligible individual factor.

**Results:**

The searches identified 10,243 database records and 15 additional records through complementary search methods. After duplicate removal and screening, 17 studies met the eligibility criteria. Fifteen examined individual characteristics in relation to occlusion-pressure outcomes, whereas two examined baseline participant characteristics in relation to subsequent morphological or functional responses to BFR. Limb circumference and resting blood pressure were the most commonly examined factors and showed the most consistent associations with AOP/LOP. Evidence for sex and limb composition was more variable. Cuff width, body position, inflation procedures, and measurement devices also affected the conditions under which AOP/LOP was determined, although these were considered methodological factors rather than individual characteristics.

**Conclusions:**

Limb size and blood pressure appear to be the individual characteristics most consistently related to AOP/LOP, but substantial methodological heterogeneity makes comparisons across studies difficult. There is still little evidence showing whether individual characteristics influence physiological, functional, efficacy, or safety outcomes once BFR is applied. Future studies should combine individualized determination of AOP with prespecified interaction, stratified, or predictive analyses to establish whether specific participant characteristics meaningfully influence subsequent responses to BFR.

## 1 Introduction

Blood flow restriction (BFR) training, also known as occlusive training or Kaatsu training (Patterson et al., 2019), involves the partial occlusion of arterial inflow and full occlusion of venous outflow in the target limb during exercise (Chulvi-Medrano et al., 2023), typically achieved using pneumatic tourniquet cuff inflated to a specific pressure or by means of non-pneumatic (e.g., straps) (Hughes et al., 2025). Methodologically, BFR is often applied with low-load resistance exercise (e.g., 20-40% of one-repetition maximum) (Lixandrão et al., 2018) or even during walking (Clarkson et al., 2017) or cycling (Kilgas et al., 2022) at low intensities. The physiological aim of BFR is to elicit a hypertrophic and strength adaptation response similar to that observed with high-load resistance training, but with significantly reduced mechanical stress (Lixandrão et al., 2018). These adaptations are believed to result from a combination of metabolic stress and mechanical stimuli, which trigger specific mechanisms such as elevated systemic hormone levels and increased recruitment of fast-twitch muscle fibers (Pearson and Hussain, 2015). These factors work synergistically to enhance muscle protein synthesis by modulating intracellular signaling pathways that favor hypertrophy and/or by promoting satellite cell activation and proliferation (Pearson and Hussain, 2015).

Approaches to BFR implementation vary in terms of cuff type (Loenneke et al., 2012b) and pressure (often determined as a percentage of limb occlusion pressure) (Clarkson et al., 2020), cuff width (Jessee et al., 2016), exercise intensity and limb occlusion pressure (Fatela et al., 2016), volume and frequency (Loenneke et al., 2012b), and inter-set rest periods (Yasuda et al., 2013), and the determination of arterial occlusion pressure (AOP), the minimum pressure required to fully occlude arterial blood flow, typically expressed as a percentage for BFR application (Souza et al., 2025). AOP is a pivotal parameter, as it directly influences the degree of occlusion and subsequent physiological responses, yet it varies significantly across individuals due to differences in limb size, vascular properties, and body position (Sieljacks et al., 2018). Research has explored regulated versus unregulated cuff pressure systems, which monitor and adjust pressure during exercise to maintain consistent occlusion (Clarkson et al., 2024). However, the extent to which individual characteristics, including sex, limb circumference, body composition, and vascular or hemodynamic factors, influence AOP remains unclear. Measurement conditions, such as cuff characteristics and body position, may also affect the AOP obtained and the associations observed with individual factors (Jessee et al., 2016; Tafuna’i et al., 2021). These methodological influences should therefore be considered separately from characteristics that are specific to the participant.

While the methodological aspects of BFR training, such as cuff type, width, and pressure, as well as exercise intensity and frequency, have been widely examined and synthesized in systematic reviews and meta-analyses (Clarkson et al., 2020; Chang et al., 2023; Rolnick et al., 2023), less attention has been given to how individual characteristics influence AOP and, consequently, BFR outcomes. Biological and anatomical variability can affect the physiological response to BFR. These individual differences may interact with methodological parameters, potentially altering both the acute and chronic adaptations. Biological and anatomical variability can alter the pressure required for effective occlusion, impacting metabolic stress and training adaptations. For instance, larger limb circumferences or higher muscle mass may necessitate higher AOP, while variations in vascular compliance or fat mass can reduce occlusion efficiency, potentially leading to suboptimal training stimuli or increased risk of adverse effects like nerve compression (Walden et al., 2024). These individual differences interact with methodological parameters, potentially explaining inconsistencies in BFR outcomes across studies. Importantly, standardized AOP protocols often fail to account for such variability, which may compromise safety by applying excessive pressure or reduce efficacy by under-occluding, highlighting the need for personalized AOP determination (Evin et al., 2021).

Despite growing research on individual-level influences (Rodrigues Neto et al., 2018; Evin et al., 2021), no comprehensive synthesis has mapped how AOP is influenced by participant characteristics or how these factors are methodologically addressed in BFR studies. A scoping review is a timely approach to systematically chart existing evidence, identify knowledge gaps, and inform future research (Peters et al., 2015). By focusing on AOP predictors, this review aims to enhance the understanding of personalized BFR protocols, ensuring both efficacy and safety in diverse populations.

Accordingly, this scoping review aimed to identify the individual characteristics that have been quantitatively examined in relation to AOP/LOP or individualized responses to BFR, and to describe how these relationships have been studied. Specifically, we sought to: (i) identify demographic, anthropometric, morphological, body-composition, and hemodynamic or vascular factors assessed in relation to AOP/LOP or BFR responses; (ii) summarize the analytical approaches used to examine these relationships, including association, prediction, stratified, and effect-modification analyses; (iii) describe the methodological conditions under which AOP/LOP was assessed, including cuff characteristics, measurement device, inflation procedure, and body position; and (iv) highlight remaining gaps in the literature, particularly whether characteristics associated with AOP/LOP also influence subsequent physiological, functional, efficacy, or safety responses to BFR.

## 2 Methods

This scoping review was conducted and reported in accordance with the Preferred Reporting Items for Systematic Reviews and Meta-Analyses Extension for Scoping Reviews (PRISMA-ScR) (Tricco et al., 2018). The review protocol was prospectively registered on the Open Science Framework (https://osf.io/osf.io/f96wd/; Date: 23/07/2025).

### 2.1 Eligibility criteria

Eligibility criteria were defined using the Population–Concept–Context (PCC) framework recommended for scoping reviews (Munn et al., 2018). The Population included human participants of any age, sex, health status, or training background undergoing assessment of AOP/LOP or application of BFR. The Concept concerned the quantitative relationship between an individual characteristic and AOP/LOP, pressure prescription, or a physiological, functional, efficacy, or safety response to BFR. The Context was unrestricted and included laboratory, sport, rehabilitation, and clinical settings, as well as pneumatic and non-pneumatic forms of BFR when the relevant relationship could be examined.

For eligibility purposes, an individual characteristic was defined as a participant attribute that was present or measured independently of the outcome being studied. These characteristics included demographic factors, such as age and sex; anthropometric and morphological measures, including limb circumference and limb dimensions; body-composition variables, such as muscle and adipose tissue measures; hemodynamic and vascular measures, including systolic and diastolic blood pressure, mean arterial pressure, and vascular indices; and baseline clinical or training characteristics when these were specifically examined in relation to the outcome.

Studies were eligible only when at least one individual characteristic was formally included in the analysis as a predictor in a regression or other predictive model, a variable in a correlation or association analysis, a basis for an explicit between-group or stratified comparison, or an interaction, moderator, or other effect-modification term for AOP/LOP or a BFR-related response. Studies were therefore not considered eligible when participant characteristics were reported descriptively only, when recruitment was limited to a particular clinical or demographic group, when BFR was prescribed as a percentage of individualized AOP/LOP without further analysis, or when efficacy or safety outcomes were reported without examining their relationship with an individual characteristic.

Cuff width and type, device characteristics, inflation procedures, body position, and other application or measurement parameters were treated as methodological factors rather than individual characteristics. These variables were extracted to describe the conditions under which associations with individual factors were assessed, but they were not sufficient on their own to meet the eligibility criteria. Studies examining only methodological influences, general BFR efficacy, or adverse events without an eligible analysis of an individual characteristic were excluded.

### 2.2 Information sources

A systematic search strategy was employed to identify all relevant published and unpublished literature. The search was not restricted by publication date, ensuring a broad mapping of the available evidence.

The following electronic databases were systematically searched on July 30, 2025: PubMed/MEDLINE, Web of Science (Core Collection), and Scopus. These databases were chosen for their extensive coverage of biomedical, health, sport, and exercise science literature, which were pertinent to the review’s scope.

### 2.3 Search

The strategy combined controlled vocabulary (e.g., Medical Subject Headings [MeSH] terms for PubMed) and keywords related to BFR training, individual characteristics, and training outcomes. Boolean operators (AND, OR) and truncation symbols (*) were used to broaden or narrow the search as appropriate. The search was iterative, with preliminary searches informing the refinement of terms.

The full PubMed search strategy is provided below and was adapted for the syntax and controlled vocabulary of Web of Science and Scopus while retaining the same core concepts. No date limits were applied. The specified search is shown below:

[Title/Abstract] “blood flow restrict*” OR “BFR” OR “arterial occlusion pressure” OR

“AOP” OR “ ischemi* training “ OR “ischemi* exercise” OR “Kaatsu” OR “Blood Flow Occlusion” OR Tourniquet* OR cuff* OR “occlusive train*” OR “limb occlusion press*” OR “vascular occlusion”

AND

[Title/Abstract] individual* OR characteristic* OR personaliz* OR sex* OR gender OR male* OR female* OR men OR man OR women OR woman OR age OR “limb circumferen*” OR anatomical* OR anthropometr* OR vascular* OR “blood pressure*” OR race OR ethnic* OR “body composit*” OR variab* OR “position*” OR “body segment*”

### 2.4 Selection of sources of evidence

Full-text eligibility was assessed independently by two authors using the criteria described above. A report was retained only when at least one eligible individual characteristic was quantitatively examined in relation to an occlusion-pressure outcome or a subsequent BFR-related response. Studies that reported participant characteristics descriptively only, or that evaluated general BFR efficacy, safety, or adverse events without an eligible individual-characteristic analysis, were excluded. Disagreements were resolved through discussion and consensus, with consultation of a third author when required. The operational clarification of this eligibility threshold was documented as an amendment to the registered review protocol.

### 2.5 Data charting process

Data charting was performed systematically using an *ad-hoc* form designed to extract relevant information pertinent to the scoping review’s objectives. The data charting form was developed collaboratively by the authors, drawing directly from the review’s objectives and the anticipated types of information present in the literature.

Prior to the formal data charting, a pilot test was conducted. Two authors independently charted data from a random sample of 3 included studies using the preliminary form. This pilot phase allowed for the identification of ambiguities, redundancy, or missing fields in the form, leading to its refinement and finalization. This calibration process ensured consistency in data extraction across authors.

Following the pilot, data charting for all included studies was conducted by two authors. Each author extracted data onto their respective forms. Discrepancies in the charted data were resolved through discussion and consensus between the two authors.

### 2.6 Data items

The initial set of data items captured study identification and characteristics, including the first author’s name, the year of publication, the country where the research was conducted, and the study’s design. We also extracted the primary aim or purpose of the study as it pertained to BFR training effect and individual characteristics.

For each included study, we recorded the individual characteristic evaluated, its analytical role (predictor, correlate, stratification factor, or effect modifier), the outcome to which it was related, the statistical approach used, and the direction and magnitude of the reported relationship where available. For downstream BFR responses, morphological, functional, efficacy, tolerability, or safety outcomes were extracted only when they were explicitly analyzed in relation to an eligible individual characteristic.

Methodological variables that could influence occlusion-pressure determination (including cuff width and type, measurement device, inflation procedure, cuff placement, body position, and measurement site) were extracted separately as contextual factors and were not considered individual characteristics.

### 2.7 Critical appraisal of individual sources of evidence

Critical appraisal was performed independently by two reviewers using JBI critical appraisal tools selected according to the analytical question and study design. The 15 studies contributing to the occlusion-pressure domain were appraised using the 2020 JBI Critical Appraisal Checklist for Analytical Cross Sectional Studies (Barker et al., 2023). This eight-item checklist was applied specifically to the participant-characteristic association or prediction analysis because the review question concerned relationships between participant-level characteristics and occlusion-pressure outcomes rather than the causal effect of an intervention. Accordingly, procedural randomization of cuff width, limb, inflation sequence, or body position was treated as a methodological feature of data collection and did not result in classification of these studies as randomized intervention trials.

Several studies also developed or evaluated multivariable equations intended to explain or predict occlusion pressure. Because the included evidence ranged from association analyses to prediction-equation development rather than representing a homogeneous set of formal clinical prediction-model studies, model-specific methodological features were evaluated descriptively in addition to the JBI appraisal. These included definition and measurement of candidate predictors, handling of repeated observations, variable selection and model specification, adjustment for relevant covariates, and the use of internal or external validation. These considerations were used to qualify interpretation of the models and were not converted into an additional numerical score or overall category.

Barbalho et al. (2019) and Ogawa et al. (2021) were appraised using the 2020 JBI Critical Appraisal Checklist for Randomized Controlled Trials (Tufanaru et al., 2020), comprising 13 appraisal items, because their principal study designs involved randomized allocation of BFR exposure. Case reports and studies examining general efficacy, safety, or methodological effects without an eligible participant-characteristic analysis were excluded during eligibility assessment and therefore did not undergo critical appraisal as included evidence.

For both JBI instruments, individual items were judged as Yes, No, Unclear, or Not applicable. No numerical quality score or generic overall low, moderate, serious, or high risk-of-bias category was derived from these judgments. Instead, item-level findings and model-specific limitations were summarized descriptively and incorporated into interpretation of the evidence. The two reviewers agreed on 97% of item-level judgments before consensus. Disagreements were resolved through discussion, with consultation of a third reviewer when consensus could not be reached. Complete item-level appraisal decisions and model-specific considerations are provided in **Supplementary Table 2**.

### 2.8 Synthesis of results

A descriptive narrative summary was developed to present the key findings from the data charting process. The summary highlighted research gaps and inconsistencies in the current literature regarding how individual characteristics were accounted for in BFR protocols, which was informed by the critical appraisal and the identified variations in methodological approaches and reported findings. The narrative also incorporated reports from the critical appraisal, discussing the methodological strengths and limitations of the included studies as they related to the investigation of individual characteristics.

AOP and LOP were mapped within a broader complete-occlusion pressure domain only when the endpoint represented disappearance of arterial pulsation or blood flow. Measures representing partial rather than complete restriction, such as the pressure required to reduce blood flow by 60%, were synthesized separately. These constructs were therefore mapped together where conceptually appropriate but were not assumed to be technically interchangeable.

An evidence gap map was constructed at the study level to show the distribution of the eligible evidence across the principal individualization questions addressed by the review. Domains were defined from the prespecified review objectives and the variables captured during data charting. These comprised: (i) participant-level characteristics examined in relation to complete occlusion pressure, including limb size, blood-pressure or other hemodynamic variables, sex, body composition or adiposity, age, and race or ethnicity; (ii) participant characteristics examined in relation to partial-flow restriction pressure; (iii) studies testing whether methodological conditions modified the relationship between participant characteristics and occlusion pressure; (iv) baseline participant characteristics examined in relation to downstream morphological, functional, efficacy, or safety responses to BFR; (v) evidence from clinical populations; and (vi) higher-level analytical gaps, including formal participant-characteristic × treatment interaction analyses, participant-level prediction of BFR-related adverse events, and external validation of occlusion-pressure prediction models.

Each eligible study was assigned to every domain to which it contributed; therefore, domain counts were not mutually exclusive and were not interpreted as numbers of unique studies across categories. The map was descriptive rather than quantitative: the number of contributing studies indicated the extent to which a question had been investigated, whereas the accompanying evidence-status field incorporated consistency of findings, methodological heterogeneity, and limitations identified during critical appraisal. Areas with several studies but inconsistent findings or substantial methodological limitations were distinguished from sparsely investigated domains and from domains in which no eligible evidence was identified. The map was therefore intended to characterize the location and nature of evidence gaps rather than to rank the clinical importance or certainty of individual characteristics.

## 3 Results

### 3.1 Study selection

The database searches identified 10,243 records: 2,222 from PubMed/MEDLINE, 3,000 from Scopus, and 5,021 from Web of Science. An additional 15 records were identified through complementary search methods, resulting in 10,258 records before duplicate removal. After removing 8,878 duplicates, 1,380 records were screened by title and abstract. Of these, 1,347 were excluded, and 33 full-text reports were assessed for eligibility. Sixteen reports were excluded after full-text assessment, leaving 17 studies for the final synthesis (**Figure 1**).

**Figure 1:**
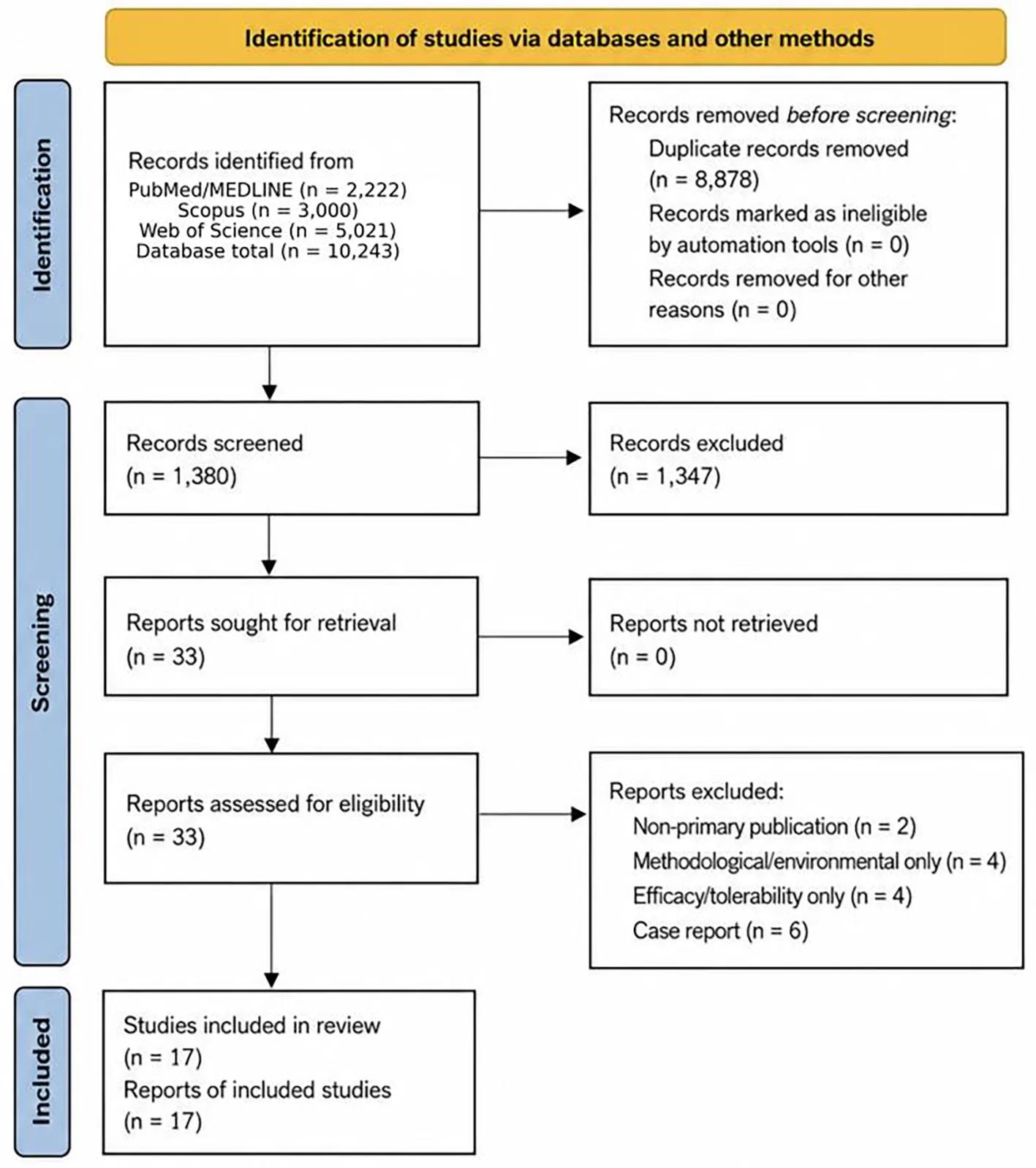
PRISMA flow diagram of study identification, screening, eligibility, and inclusion.

The included evidence covered two main areas. Fifteen studies examined associations between individual characteristics and occlusion-pressure outcomes, whereas two investigated whether baseline participant characteristics were related to subsequent morphological or functional responses to BFR. Studies that reported only general efficacy, tolerability, or adverse events without formally examining the role of an individual characteristic were not included.

The 16 reports excluded at the full-text stage fell into four main categories. Two were non-primary publications and were excluded because the review was restricted to primary human research (McEwen et al., 2019; de Queiros et al., 2024). Four primary studies focused on methodological or environmental factors, including body position, cuff-related hemodynamic conditions, or reduced-gravity analogues, without statistically examining an eligible individual characteristic (Hughes et al., 2018; Mouser et al., 2017; Nakajima et al., 2008; Swain et al., 2026). A further four studies assessed BFR efficacy, tolerability, or rehabilitation outcomes but did not test whether participant characteristics predicted or modified these responses (Cancio et al., 2019; Hughes et al., 2019; Mattar et al., 2014; Scott et al., 2017). The remaining six reports were case reports describing clinical applications or adverse events and did not include an eligible analysis of participant characteristics (Clark and Manini, 2017; Cohen et al., 2021; Iversen and Røstad, 2010; Kohlbrenner et al., 2021; Noto et al., 2017; Tabata et al., 2016).

### 3.2 Study characteristics

The 17 included studies had sample sizes ranging from 20 to 249 participants. All included both males and females, although the proportion of each sex varied considerably across studies. Most of the evidence on occlusion pressure came from healthy young adults, recreationally active participants, university students, and trained athletes. Clinical populations were represented only in the two studies that examined subsequent responses to BFR, involving older adults in intensive care and patients undergoing rehabilitation after cardiovascular surgery. The demographic, methodological, and analytical characteristics of each study are summarized in **Table 1**.

**Table 1:** Demographic, methodological, and analytical characteristics of the included studies.

Study	N (sex)	Population and age	Study design	Evidence domain/outcome construct	Participant characteristic(s) evaluated	Analytical approach	AOP/LOP/BFR method or intervention
(Barnett et al., 2016)	31 (19 M/12 F)	Recreationally active adults; 23 ± 3 y	Analytical cross-sectional with acute repeated AOP measurement	AOP; right upper limb	Arm circumference; brachial SBP	Hierarchical linear regression	5-cm pneumatic cuff; radial-artery Doppler; standing; exercise at 40% resting AOP.
(Borges et al., 2018)	62 (31 M/31 F)	Healthy physically active university students; M 21.7 ± 2.3, F 22.0 ± 2.0 y	Analytical cross-sectional	AOP; dominant upper limb	Sex; arm circumference; SBP	Multiple linear regression	6-cm pneumatic cuff; radial-artery Doppler; seated.
(Brown et al., 2018)	20 (10 M/10 F)	Well-trained athletes; 22.2 ± 2.8 y	Randomized crossover measurement study	AOP; upper and lower limbs	Sex; SBP; anthropometry; DXA-derived limb composition	Pearson correlations and multiple regression	Doppler-confirmed AOP; 6/13-cm upper-limb and 13/18-cm lower-limb cuffs; cuff size and limb tested in crossover fashion.
(Evin et al., 2021)	42 (25 M/17 F)	Healthy adults; 25.8 ± 5.2 y	Repeated-measures analytical study	LOP; bilateral lower limbs	Pulse pressure; thigh circumference; SBP; DBP; MAP; sex	Linear mixed-effects models and variance-component analysis	12-cm contoured cuff; automated PTS II system; supine; repeated across legs and days.
(Hunt et al., 2016)	47 analyzed (24 M/23 F)	Healthy adults; 21 ± 3 y	Analytical cross-sectional	Pressure required for 60% BFR; upper and lower limbs	BP; BMI; limb circumference; adipose thickness; muscle–bone CSA; vessel characteristics	Hierarchical and stepwise regression	Doppler blood-flow measurement in popliteal and brachial arteries; 13-cm thigh and 11-cm arm cuffs; target = 60% reduction in blood flow.
(Jessee et al., 2016)	249 (102 M/147 F)	Healthy adults; 18–35 y	Analytical cross-sectional with repeated cuff-width measurements	AOP; right upper limb	Arm circumference; brachial SBP/DBP; arm length; sex; race	Hierarchical linear regression and repeated-measures comparisons	Radial-artery Doppler; 5-, 10- and 12-cm cuffs; standing.
(Karanasios et al., 2022)	42 (29 M/13 F)	Healthy adults; 28.1 ± 7.7 y	Randomized repeated-measures reliability/association study	AOP; dominant upper limb	Arm circumference; SBP; DBP	Multiple and pairwise linear regression	7-cm manual cuff with handheld Doppler; randomized supine, seated and standing assessments.
(Loenneke et al., 2012a)	116 (53 M/63 F)	Healthy adults; 22 ± 3 y	Analytical cross-sectional with repeated cuff-width measurements	AOP; lower limb	Thigh circumference; muscle/fat CSA; BP; ankle–brachial index	Hierarchical linear regression	Posterior-tibial Doppler; 5-cm and 13.5-cm thigh cuffs; supine.
(Loenneke et al., 2015)	171 (52 M/119 F)	Healthy adults; 21 ± 3 y	Analytical cross-sectional	AOP; upper and lower limbs	Arm/thigh circumference; SBP/DBP; muscle thickness; fat thickness	Hierarchical linear regression	5-cm cuffs; radial and posterior-tibial Doppler; supine.
(de Queiros et al., 2024)	51 (25 M/26 F)	Healthy adults; median 24 y (IQR 22–27)	Cross-sectional repeated-measures study	LL-AOP; right lower limb	SBP; DBP; thigh circumference; sex	Hierarchical linear regression	Posterior-tibial Doppler; 11- and 18-cm cuffs; supine, seated and standing.
(Sieljacks et al., 2018)	24 (14 M/10 F)	Healthy adults; 32 ± 13 y	Randomized repeated-measures study	AOP; lower limb	Thigh circumference; DBP; SBP; skinfold	Multiple linear regression	Posterior-tibial Doppler; 13-cm and 6-cm cuffs; supine and seated.
(Tafuna’i et al., 2021)	35 (16 M/19 F)	Physically active healthy adults; 23.3 ± 3.5 y	Analytical cross-sectional/repeated-leg study	AOP; dominant and non-dominant lower limbs	Sex; leg circumference; SBP; DBP; MAP; skinfold; age	Group comparisons and regression	Superficial-femoral Doppler; 10-cm cuff; semi-reclined.
(Vehrs et al., 2023)	40 (20 M/20 F)	Physically active healthy adults; 19–26 y	Repeated-measures methodological/association study	AOP; bilateral lower limbs	Sex; thigh circumference; SBP; DBP; MAP; skinfold; thigh volume	Mixed-model ANOVA and predictor analysis	Superficial-femoral Doppler; 10-cm cuff; continuous and incremental inflation protocols; semi-reclined.
(Walden et al., 2024)	22 (12 M/10 F)	Physically active adults; 26 ± 4 y	Observational cross-sectional	AOP; dominant lower limb	DXA-derived muscle/fat/bone mass; limb circumference; SBP as covariate	Pearson correlations with covariate adjustment	Posterior-tibial Doppler; 10-cm cuff; supine; three AOP measurements.
(Rodrigues Neto et al., 2018)	156 (76 M/80 F)	Healthy adults; 23.9 ± 3.7 y	Randomized crossover measurement study	BFR point; upper and lower limbs	Sex	Between-sex and repeated-position/segment comparisons	Vascular Doppler with standard sphygmomanometer cuffs; upper and lower limbs; supine, seated and standing.
(Barbalho et al., 2019)	20 (17 M/3 F)	Older ICU patients in coma; 66 ± 4.3 y	Within-patient randomized trial	Downstream BFR response: muscle wasting	Baseline body mass	Pearson correlation of baseline characteristic with change	Unilateral lower-limb BFR added to passive mobilization; cuff pressure set relative to anterior-tibial arterial pressure.
(Ogawa et al., 2021)	21 (18 M/3 F)	Patients after cardiovascular surgery; 69.6 ± 12.6 y	Pilot randomized controlled trial	Downstream BFR response: muscle size and physical function	Baseline MTH, SMI, walking speed, knee-extensor strength	Pearson correlations between baseline level and 3-month change	KAATSU NANO; bilateral lower-limb resistance training; cuff pressure progressed from 100 to 160–200 mmHg.

AOP, arterial occlusion pressure; BFR, blood flow restriction; CSA, cross-sectional area; DBP, diastolic blood pressure; DXA, dual-energy X-ray absorptiometry; LL-AOP, lower-limb arterial occlusion pressure; LOP, limb occlusion pressure; MAP, mean arterial pressure; MTH, anterior mid-thigh thickness; SBP, systolic blood pressure; SMI, skeletal muscle mass index.

Considerable methodological variation was evident across the studies assessing occlusion pressure. Twelve studies referred to arterial occlusion pressure (AOP or LL-AOP), generally defined as complete arterial occlusion and determined by the disappearance of distal arterial pulsation or blood flow using Doppler assessment. Evin et al. (2021) reported limb occlusion pressure (LOP) measured with an automated personalized tourniquet system. Hunt et al. (2016), in contrast, examined the cuff pressure required to reduce arterial blood flow by 60%, representing partial rather than complete occlusion. Rodrigues Neto et al. (2018) used the term BFR point to describe the pressure at which the auscultatory pulse was no longer detected. For mapping purposes, these outcomes were grouped within a broader occlusion-pressure domain, while recognizing that they are not technically equivalent. Differences in measurement site, cuff width, device, inflation procedure, and body position are detailed in **Table 1** and were taken into account when interpreting variation across studies.

### 3.3 Critical appraisal by study design

For the 15 studies examining occlusion-pressure outcomes, critical appraisal focused on the participant-characteristic association or prediction analysis using the 2020 JBI Critical Appraisal Checklist for Analytical Cross Sectional Studies. Randomization of cuff order, limb, body position, or other measurement conditions was treated as a procedural feature rather than as intervention randomization. Features relevant to prediction-equation development were additionally considered descriptively, including predictor definition, handling of repeated observations, model specification, adjustment for relevant covariates, and internal or external validation. Barbalho et al. (2019) and Ogawa et al. (2021) were appraised using the 2020 JBI Critical Appraisal Checklist for Randomized Controlled Trials.

Across the occlusion-pressure studies, eligibility criteria and study settings were generally well described, and pressure outcomes were typically assessed using objective vascular measures. The most common limitations were convenience sampling, limited control of potential confounding, relatively small samples in several studies, and the development and evaluation of prediction equations within the same dataset. None of the proposed equations underwent external validation. In studies with repeated measurements, statistical dependence between limbs, cuff conditions, or body positions was addressed using mixed or repeated-measures approaches in some cases, but this was not consistent across studies.

The two randomized studies used objective morphological or functional outcomes and reported random allocation, although both had small samples. Barbalho et al. (2019) randomized limbs within participants and included blinded data analysis, but blinding of treating personnel was not possible and allocation concealment was insufficiently described. Ogawa et al. (2021) conducted a pilot parallel-group randomized trial with prospective safety monitoring; however, reporting of allocation concealment and blinding was incomplete. In addition, the correlations between baseline characteristics and subsequent changes were exploratory and should not be interpreted as formal analyses of treatment-effect modification. **Table 2** summarizes the appraisal approach applied to each study design and the main methodological limitations relevant to interpretation.

**Table 2:** Critical appraisal summary for the included evidence.

Design group	Studies	JBI tool	Main methodological strengths	Main limitations relevant to interpretation
Occlusion-pressure association/prediction analyses (n = 15)	Barnett; Borges; Brown; Evin; Hunt; Jessee; Karanasios; Loenneke (2012, 2015); de Queiros; Sieljacks; Tafuna’i; Vehrs; Walden; Rodrigues Neto	JBI Critical Appraisal Checklist for Analytical Cross Sectional Studies; model-specific prediction features considered descriptively	Generally clear eligibility criteria and participant descriptions; objective vascular endpoints in most studies; inferential analyses usually matched the stated association/prediction questions.	Convenience samples; several small samples; confounder selection and adjustment were inconsistent; equations were commonly derived and evaluated in the same sample; no external validation; repeated observations and device ceiling effects were not handled uniformly.
Within-patient randomized trial (n = 1)	Barbalho et al. (2019)	JBI Critical Appraisal Checklist for Randomized Controlled Trials	Randomized limb allocation; concurrent within-patient control; objective ultrasound outcome; blinded data analysis.	Small clinical sample; treating personnel not blinded; allocation concealment insufficiently detailed; participant-characteristic correlation was exploratory and not a prespecified effect-modification analysis.
Parallel-group pilot randomized trial (n = 1)	Ogawa et al. (2021)	JBI Critical Appraisal Checklist for Randomized Controlled Trials	Random group allocation; repeated objective morphological/functional measurements; prospective monitoring of clinical safety markers.	Small pilot sample; allocation concealment and blinding not fully reported; exploratory baseline-to-change correlations were underpowered for interaction testing and susceptible to regression-to-the-mean/mathematical-coupling effects.

### 3.4 Participant characteristics associated with occlusion pressure

Limb size was the most frequently investigated participant characteristic (**Table 3**). Arm or thigh circumference contributed to AOP or LOP in most studies in which it was modeled, although the magnitude of its contribution depended on cuff width, measurement site, and the specific occlusion construct. Barnett et al. (2016) reported that arm circumference and brachial SBP together explained 69% of standing upper-limb AOP variance, with circumference contributing the largest unique proportion. Borges et al. (2018) similarly found arm circumference, SBP, and sex to be significant predictors of seated upper-limb AOP. In the lower limb, Sieljacks et al. (2018); Tafuna’i et al. (2021); Vehrs et al. (2023), and Evin et al. (2021) identified thigh or leg circumference as an important correlate or predictor of complete occlusion pressure.

**Table 3:** Findings for participant characteristics and individualized BFR-related outcomes.

Study	Outcome construct	Characteristic(s)	Analysis	Main finding	Specificity
Barnett et al. (2016)	AOP; right upper limb	Arm circumference; brachial SBP	Hierarchical linear regression	Arm circumference and brachial SBP explained 69% of standing AOP variance; arm circumference contributed the largest unique share. AOP increased immediately after exercise.	Occlusion-pressure determinant/association
Borges et al. (2018)	AOP; dominant upper limb	Sex; arm circumference; SBP	Multiple linear regression	Arm circumference, SBP and sex were significant predictors; the model explained 42% of seated AOP variance.	Occlusion-pressure determinant/association
Brown et al. (2018)	AOP; upper and lower limbs	Sex; SBP; anthropometry; DXA-derived limb composition	Pearson correlations and multiple regression	Sex and SBP explained 77–83% of upper-limb and 61–63% of lower-limb AOP variance. Adding anthropometry and DXA variables increased explained variance to as high as 91% and 87%, respectively.	Occlusion-pressure determinant/association
Evin et al. (2021)	LOP; bilateral lower limbs	Pulse pressure; thigh circumference; SBP; DBP; MAP; sex	Linear mixed-effects models and variance-component analysis	Pulse pressure and thigh circumference were positively associated with LOP; SBP, DBP, MAP and sex were not. Forty-seven percent of LOP variance was between participants.	Occlusion-pressure determinant/association
Hunt et al. (2016)	Pressure required for 60% BFR; upper and lower limbs	BP; BMI; limb circumference; adipose thickness; muscle–bone CSA; vessel characteristics	Hierarchical and stepwise regression	Determinants differed from complete occlusion. MAP and adipose thickness showed the largest independent relationships with lower- and upper-body partial-occlusion pressure; regression models explained up to 48% of variance, leaving substantial unexplained variability.	Occlusion-pressure determinant/association
Jessee et al. (2016)	AOP; right upper limb	Arm circumference; brachial SBP/DBP; arm length; sex; race	Hierarchical linear regression and repeated-measures comparisons	Arm circumference was the strongest contributor across cuff widths, with brachial SBP also important. Sex remained relevant in multivariable models and race was associated with AOP after adjustment.	Occlusion-pressure determinant/association
Karanasios et al. (2022)	AOP; dominant upper limb	Arm circumference; SBP; DBP	Multiple and pairwise linear regression	Arm circumference and SBP were consistently associated with AOP across supine, seated and standing positions; DBP did not add consistent explanatory value.	Occlusion-pressure determinant/association
Loenneke et al. (2012a)	AOP; lower limb	Thigh circumference; muscle/fat CSA; BP; ankle–brachial index	Hierarchical linear regression	Wider cuffs required lower AOP. Limb size and composition accounted for a substantial proportion of AOP variability; models explained more variance with the wide than the narrow cuff.	Occlusion-pressure determinant/association
Loenneke et al. (2015)	AOP; upper and lower limbs	Arm/thigh circumference; SBP/DBP; muscle thickness; fat thickness	Hierarchical linear regression	Arm circumference and SBP were the dominant field predictors of upper-limb AOP; thigh circumference and SBP contributed most to lower-limb AOP. Field models explained about 60% of upper-limb and 49% of lower-limb variance.	Occlusion-pressure determinant/association
de Queiros et al. (2024)	LL-AOP; right lower limb	SBP; DBP; thigh circumference; sex	Hierarchical linear regression	The dominant predictor depended on cuff width: SBP contributed most with the 18-cm cuff, whereas thigh circumference was more influential with the 11-cm cuff. Body position also materially changed LL-AOP.	Occlusion-pressure determinant/association
Sieljacks et al. (2018)	AOP; lower limb	Thigh circumference; DBP; SBP; skinfold	Multiple linear regression	Thigh circumference was the strongest predictor with both cuffs. DBP explained additional variance with the wide cuff; SBP and skinfold did not contribute consistently. Body position strongly changed AOP.	Occlusion-pressure determinant/association
Tafuna’i et al. (2021)	AOP; dominant and non-dominant lower limbs	Sex; leg circumference; SBP; DBP; MAP; skinfold; age	Group comparisons and regression	Absolute AOP was higher in men. After leg circumference entered the model, SBP, DBP, MAP, skinfold, age and sex were not independent predictors; leg circumference was the principal predictor.	Occlusion-pressure determinant/association
Vehrs et al. (2023)	AOP; bilateral lower limbs	Sex; thigh circumference; SBP; DBP; MAP; skinfold; thigh volume	Mixed-model ANOVA and predictor analysis	No sex difference in AOP was observed. Thigh circumference was the only significant participant-level predictor; BP, skinfold thickness and thigh volume did not add significant explanatory value.	Occlusion-pressure determinant/association
Walden et al. (2024)	AOP; dominant lower limb	DXA-derived muscle/fat/bone mass; limb circumference; SBP as covariate	Pearson correlations with covariate adjustment	Lower-limb muscle mass showed a moderate negative relationship with AOP, whereas limb circumference had the weakest relationship with AOP among the reported composition measures.	Occlusion-pressure determinant/association
Rodrigues Neto et al. (2018)	BFR point; upper and lower limbs	Sex	Between-sex and repeated-position/segment comparisons	BFR point differed between sexes in several limb/position comparisons, demonstrating that sex was directly evaluated against the occlusion outcome rather than used only descriptively.	Occlusion-pressure determinant/association
Barbalho et al. (2019)	Downstream BFR response: muscle wasting	Baseline body mass	Pearson correlation of baseline characteristic with change	Initial body mass was not associated with quadriceps muscle-thickness change in the BFR limb (r = 0.17, p = 0.45). Intervention duration, rather than body mass, was associated with the magnitude of wasting.	Exploratory baseline-to-response association; not formal moderation
Ogawa et al. (2021)	Downstream BFR response: muscle size and physical function	Baseline MTH, SMI, walking speed, knee-extensor strength	Pearson correlations between baseline level and 3-month change	In the KAATSU group, lower baseline walking speed was associated with greater improvement (r = −0.71, p = 0.015); baseline MTH and SMI showed moderate negative, non-significant associations (both r = −0.57, p = 0.07), while baseline knee-extensor strength was not associated with change (r = −0.18, p = 0.59).	Exploratory baseline-to-response association; not formal moderation

Blood-pressure variables also contributed, but their relevance differed across protocols. SBP was informative in upper-limb models from Barnett et al. (2016); Borges et al. (2018); Brown et al. (2018); Karanasios et al. (2022), and Loenneke et al. (2015). Evin et al. (2021) identified pulse pressure, rather than SBP, DBP, or MAP, as independently associated with LOP. Sieljacks et al. (2018) found that DBP added explanatory value when a wide lower-limb cuff was used. de Queiros et al. (2024) showed that the relative contribution of SBP and thigh circumference changed with cuff width: SBP was more influential with the 18-cm cuff, whereas thigh circumference contributed more strongly with the 11-cm cuff. These findings indicate that the relationship between participant characteristics and occlusion pressure is conditional on the measurement configuration rather than fixed across devices and protocols.

Sex-related findings were not uniform. Rodrigues Neto et al. (2018) and Tafuna’i et al. (2021) reported sex differences in occlusion pressure in several comparisons. Jessee et al. (2016) found that sex retained explanatory value after accounting for other individual characteristics, whereas Vehrs et al. (2023) reported no sex difference and identified thigh circumference as the only significant participant-level predictor. Evin et al. (2021) likewise found no independent association between sex and LOP. Body-composition measures provided additional information in selected studies. Brown et al. (2018) improved AOP prediction after adding surface anthropometry and DXA-derived measures, while Walden et al. (2024) reported a moderate inverse relationship between lower-limb muscle mass and AOP. Hunt et al. (2016) differed from the complete-occlusion literature because it modeled pressure required for 60% BFR: MAP and adipose thickness showed the largest independent relationships, and limb circumference had a more limited role. This difference supports separate interpretation of partial-flow restriction and complete-occlusion endpoints.

### 3.5 Participant characteristics associated with downstream BFR responses

Only two studies directly examined participant-level characteristics in relation to subsequent BFR responses. In older intensive-care patients, Barbalho et al. (2019) tested whether initial body mass was associated with quadriceps muscle-thickness change. No association was observed in the BFR limb (r = 0.17, p = 0.45), while a longer intervention period was associated with greater muscle loss. In patients undergoing cardiovascular rehabilitation, Ogawa et al. (2021) examined baseline muscle size and physical function in relation to 3-month change. Within the KAATSU group, lower baseline walking speed was associated with greater improvement (r = −0.71, p = 0.015). Baseline MTH and SMI each showed moderate negative but non-significant associations with change (r = −0.57, p = 0.07), whereas baseline knee-extensor strength was not associated with strength change (r = −0.18, p = 0.59).

Neither study formally tested whether a participant characteristic modified the treatment effect using an interaction term between the characteristic and BFR exposure. The available evidence therefore supports only exploratory baseline-to-response associations and does not establish treatment-effect heterogeneity. No included study quantitatively examined whether participant characteristics predicted BFR-related adverse events or safety outcomes.

### 3.6 Evidence distribution and evidence gaps

The evidence gap map showed a marked concentration of research on participant-level determinants of occlusion pressure, with substantially less evidence addressing heterogeneity in subsequent BFR responses (**Table 4**). Limb circumference and blood-pressure or other hemodynamic variables represented the most densely populated domains. Sex and body-composition variables had also been examined in several studies, although findings were less consistent and the analytical and measurement approaches differed considerably. In contrast, race or ethnicity and age were represented by very limited direct evidence.

**Table 4:** Evidence gap map with contributors.

Evidence domain	Contributing study/studies	Evidence status and gap
Limb circumference/limb size → complete AOP or LOP	Barnett et al. (2016); Borges et al. (2018); Brown et al. (2018); Evin et al. (2021); Jessee et al. (2016); Karanasios et al. (2022); Loenneke et al. (2012a, 2015); de Queiros et al. (2024); Sieljacks et al. (2018); Tafuna’i et al. (2021); Vehrs et al. (2023); Walden et al. (2024)	Relatively extensive evidence, but the magnitude and apparent importance of limb size vary by cuff, device, position, and outcome definition; no generalizable prediction equation has been established.
Blood pressure/hemodynamic characteristics → AOP or LOP	Barnett et al. (2016); Borges et al. (2018); Brown et al. (2018); Evin et al. (2021); Hunt et al. (2016); Jessee et al. (2016); Karanasios et al. (2022); Loenneke et al. (2012a, 2015); de Queiros et al. (2024); Sieljacks et al. (2018); Tafuna’i et al. (2021); Vehrs et al. (2023); Walden et al. (2024)	Relatively extensive but heterogeneous evidence; SBP, DBP, MAP, and pulse pressure are not interchangeable, and their contributions vary across pressure constructs and measurement configurations.
Sex → occlusion pressure	Borges et al. (2018); Brown et al. (2018); Evin et al. (2021); Jessee et al. (2016); Rodrigues Neto et al. (2018); Tafuna’i et al. (2021); Vehrs et al. (2023)	Evidence present but inconsistent; sex-related differences were not uniform and were attenuated or absent after accounting for limb size or hemodynamic variables in some analyses.
Body composition/adiposity → occlusion pressure	Brown et al. (2018); Hunt et al. (2016); Loenneke et al. (2012a, 2015); Sieljacks et al. (2018); Tafuna’i et al. (2021); Vehrs et al. (2023); Walden et al. (2024)	Evidence present but heterogeneous; the available studies used non-equivalent constructs including DXA-derived measures, CSA, skinfolds, adipose thickness, and tissue thickness.
Race/ethnicity → AOP	Jessee et al. (2016)	Single-study evidence.
Age → AOP/LOP as an independent characteristic	Tafuna’i et al. (2021)	Very limited direct analysis; included samples were predominantly young adults.
Participant characteristics → partial BFR pressure rather than complete occlusion	Hunt et al. (2016)	Single-study evidence; determinants differed from complete-occlusion studies.
Methodological context modifies characteristic–pressure relationship	Brown et al. (2018); Jessee et al. (2016); Karanasios et al. (2022); Loenneke et al. (2012a); de Queiros et al. (2024); Sieljacks et al. (2018); Vehrs et al. (2023)	Cuff width, position, inflation protocol and device influence measured pressure and/or predictor strength.
Baseline anthropometry → downstream BFR response	Barbalho et al. (2019)	Single exploratory analysis; body mass was not associated with muscle-thickness change.
Baseline morphology/physical function → downstream BFR response	Ogawa et al. (2021)	Single pilot study; associations varied by outcome and were not tested as treatment-effect interactions.
Clinical populations with participant-level response analysis	Barbalho et al. (2019); Ogawa et al. (2021)	Sparse and condition-specific evidence; generalizability is limited.
Formal participant-characteristic × BFR treatment interaction/effect modification	None	No eligible evidence identified. No study formally tested whether a participant characteristic modified the treatment effect of BFR.
Participant-level predictors of BFR-related adverse events or safety outcomes	None	No eligible evidence identified. Safety-related individual susceptibility has not been quantitatively examined within the eligible literature.
External validation of occlusion-pressure prediction equations	None	No eligible evidence identified. Prediction equations were derived and, in some cases, internally evaluated, but none was validated in an independent sample.

The map also identified qualitatively different types of evidence gaps. Some domains contained multiple studies but remained methodologically heterogeneous or produced inconsistent findings, as observed for sex and body composition. Other domains were sparse, with only one or two contributing studies, including race or ethnicity, age, partial-flow restriction pressure, and participant characteristics associated with downstream BFR responses. Finally, several questions were characterized by a complete absence of eligible evidence: no study formally tested a participant-characteristic × BFR interaction to establish treatment-effect modification, no study quantitatively examined participant-level predictors of BFR-related adverse events, and no published occlusion-pressure prediction equation had undergone external validation in an independent sample. **Table 4** identifies the studies contributing to each domain and distinguishes these forms of evidence limitation.

## 4 Discussion

This scoping review mapped how individual characteristics have been examined in relation to occlusion pressure and subsequent responses to BFR. The evidence was strongly concentrated on pressure determination since 15 of the 17 included studies evaluated characteristics associated with an occlusion-pressure outcome, whereas only two examined whether baseline participant characteristics were related to later morphological or functional responses to BFR. Across the pressure studies, limb size and blood-pressure measures emerged most often as relevant correlates or model contributors, but neither operated independently of the measurement context. Sex and body-composition findings were less consistent, and age and race were rarely examined directly. Thus, the literature is better developed for explaining variation in the pressure required to achieve occlusion than for explaining why people respond differently once BFR is applied.

This distinction is important for interpreting individualization in BFR. A variable that helps explain arterial or limb occlusion pressure is not necessarily a modifier of the subsequent training or rehabilitation response. The included literature largely addressed the first question and provided very little evidence for the second. The review therefore contributes an evidence map of where individualization has actually been studied, while also showing that methodological factors such as cuff width, body position, device, and inflation procedure can alter measured pressure and the apparent importance of participant characteristics. These procedural factors are part of the measurement context rather than attributes of the individual, and separating the two is necessary if prediction equations or personalized pressure strategies are to be interpreted appropriately.

### 4.1 Individual characteristics associated with occlusion pressure

Limb circumference was the clearest evidence cluster, although the strength of the association varied across protocols. Upper-limb studies generally found arm circumference to contribute meaningfully to AOP alongside systolic blood pressure (SBP) (Barnett et al., 2016; Borges et al., 2018; Jessee et al., 2016; Loenneke et al., 2015). A similar pattern was observed in the lower limb, where thigh or leg circumference was associated with complete occlusion pressure in several studies (Evin et al., 2021; Sieljacks et al., 2018; Tafuna’i et al., 2021; Vehrs et al., 2023). This consistency should not, however, be interpreted as evidence for a single circumference-based prescription rule. The contribution of limb size changed with cuff width, limb, body position, and the pressure construct being modeled. de Queiros et al. (2024), for example, showed that the relative contribution of thigh circumference and SBP differed between cuff widths, while Jessee et al. (2016) reported cuff-specific differences in model performance. Limb circumference is therefore best viewed as a recurrent determinant whose predictive value is conditional on how occlusion pressure is measured.

Blood-pressure variables showed a similar context-dependent pattern. SBP contributed to several upper- and lower-limb models, but other hemodynamic measures were not consistently interchangeable with it. Evin et al. (2021) identified pulse pressure, rather than SBP, diastolic blood pressure, or mean arterial pressure, as independently associated with LOP, whereas Sieljacks et al. (2018) found additional explanatory value for diastolic blood pressure under a specific cuff condition. Hunt et al. (2016) also identified hemodynamic and adiposity-related factors when modeling the pressure required to reduce blood flow by 60%. That study addressed partial restriction rather than complete arterial occlusion, and its findings should not be assumed to apply directly to AOP or LOP. The broader pattern is therefore not that one blood-pressure measure is universally predictive, but that systemic pressure contributes to occlusion-pressure variability in ways that depend on the limb, cuff, endpoint, and analytical model.

Evidence for sex was more heterogeneous. Borges et al. (2018); Jessee et al. (2016); Rodrigues Neto et al. (2018), and Tafuna’i et al. (2021) reported sex-related differences or retained sex in some analyses, whereas Evin et al. (2021) and Vehrs et al. (2023) did not identify an independent sex effect. In several studies, limb size or hemodynamic variables explained more of the variation than sex itself. This makes a simple sex-specific interpretation difficult and does not support different pressure prescriptions based on sex alone. No included study evaluated menstrual-cycle phase or hormonal status as a determinant of AOP/LOP, so physiological hypotheses about these factors should remain separate from conclusions drawn from the mapped evidence.

Body-composition findings were also fragmented. Brown et al. (2018); Hunt et al. (2016), Loenneke et al. (2012a, 2015), and Walden et al. (2024) suggest that tissue composition can add information beyond gross limb circumference, but the variables and measurement approaches differed substantially. DXA-derived lean, fat, and bone measures, cross-sectional area, adipose thickness, skinfolds, and ultrasound-derived tissue thickness are not equivalent constructs. Walden et al. (2024), for example, reported an inverse relationship between lower-limb muscle mass and AOP, while other studies emphasized limb circumference or blood pressure. The evidence is therefore insufficient to identify one body-composition variable as a generalizable determinant. Age and race were even less developed: age was directly examined in very limited analyses, and race was represented principally by Jessee et al. (2016). These are gaps in the evidence rather than evidence that the characteristics are unimportant.

### 4.2 Measurement context and interpretability of association and prediction models

The methodological heterogeneity observed across the pressure studies is not merely a reporting issue since seem to be an indication of the associations being estimated. Cuff width alters the external pressure required to occlude a limb, and body position changes the hydrostatic and vascular conditions under which the measurement is made. Studies that repeated AOP across cuff widths or positions showed that both the absolute pressure and the apparent contribution of participant characteristics could change across configurations (Jessee et al., 2016; Karanasios et al., 2022; de Queiros et al., 2024; Sieljacks et al., 2018). Inflation protocol and device characteristics add further variation (Evin et al., 2021; Vehrs et al., 2023). Consequently, a regression equation developed with one cuff, device, body position, and anatomical site cannot be assumed to transport unchanged to another configuration.

The distinction among AOP, LOP, partial-flow restriction pressure, and the BFR point also matters. Most included studies used disappearance of an arterial pulse or flow signal to define complete occlusion, but Evin et al. (2021) used an automated personalized tourniquet system to determine LOP, Hunt et al. (2016) modeled the pressure required for 60% reduction in blood flow, and Rodrigues Neto et al. (2018) used the term BFR point. These outcomes are related to pressure prescription but are not technically identical. Mapping them within a broader occlusion-pressure domain is useful for showing the breadth of the literature, yet direct numerical comparison should remain cautious because the physiological endpoint and measurement procedure differ.

The critical appraisal reinforces this caution. The occlusion-pressure studies generally described participants and eligibility criteria adequately and usually relied on objective vascular endpoints, but several recurring limitations reduce confidence in the portability of the reported models. Convenience sampling was common, sample sizes were modest in a number of studies, selection and adjustment of potential confounders were inconsistent, and repeated observations from limbs, cuff conditions, or body positions were not always handled with methods that explicitly modeled within-participant dependence. Prediction equations were commonly developed and evaluated in the same dataset. Although some studies incorporated internal validation procedures, none of the proposed equations underwent external validation in an independent sample. The available models should therefore be interpreted as study-specific estimates of association or prediction rather than established tools with demonstrated transportability across populations and BFR systems.

### 4.3 Individual characteristics associated with downstream BFR responses

The evidence changed markedly once the question moved from pressure determination to response heterogeneity. Only Barbalho et al. (2019) and Ogawa et al. (2021) quantitatively related a baseline participant characteristic to a subsequent BFR response. In older intensive-care patients, baseline body mass was not associated with quadriceps muscle-thickness change in the BFR limb (Barbalho et al., 2019). In patients undergoing rehabilitation after cardiovascular surgery, lower baseline walking speed was associated with greater subsequent improvement within the KAATSU group, whereas associations for baseline muscle thickness and skeletal muscle mass index did not reach conventional statistical significance and baseline knee-extensor strength was not related to strength change (Ogawa et al., 2021). These studies broaden the individualization question beyond AOP/LOP, but they remain isolated examples in distinct clinical settings.

More importantly, neither study formally tested treatment-effect modification with a participant-characteristic-by-treatment interaction. Their analyses were exploratory baseline-to-change associations, which cannot establish that a characteristic alters the causal effect of BFR. The small samples and, particularly for baseline-to-change correlations, the potential influence of regression to the mean and mathematical coupling further limit interpretation. The critical appraisal therefore reduces confidence in using these findings to identify likely responders or to tailor clinical BFR protocols. The current evidence supports only the conclusion that downstream response heterogeneity has rarely been studied directly.

The same limitation applies to safety. No eligible study quantitatively examined whether participant characteristics predicted BFR-related adverse events. General efficacy trials and adverse-event case reports may be informative for other review questions, but without an analysis linking participant characteristics to outcome they cannot establish individualized efficacy, susceptibility, or treatment-effect heterogeneity. Their exclusion from the present synthesis is therefore methodologically important. It also means that this review cannot estimate adverse-event incidence or conclude that BFR is generally safe or effective across populations. Those questions require different eligibility criteria and synthesis methods.

### 4.4 Evidence distribution and interpretation of the evidence gap map

The evidence gap map complements the study-characteristics and findings tables by shifting the unit of interpretation from individual studies to evidence domains. It shows not only where research activity is concentrated, but also whether an apparent gap reflects inconsistent or methodologically heterogeneous evidence, sparse investigation, or a complete absence of eligible studies. Limb circumference and blood-pressure variables form the densest evidence clusters, but the studies within those clusters remain methodologically heterogeneous. Sex and body composition occupy an intermediate position: several studies are available, yet the results are inconsistent and the constructs or analytical adjustments differ. Race and age represent sparse domains with little direct investigation. These are different problems. A domain with several heterogeneous studies requires replication under standardized conditions, whereas a domain represented by one study or none at all requires basic evidence generation before consistency can be judged.

The most consequential empty areas occur downstream of pressure determination. Only two studies examined participant characteristics in relation to subsequent BFR responses, no study tested a formal treatment-by-characteristic interaction, and no included study examined participant-level predictors of BFR-related adverse events. Likewise, external validation of pressure-prediction equations was absent. The map should therefore be read as a distribution of evidence rather than a hierarchy of importance. Dense cells indicate that a question has been studied more often, not that the corresponding variable is clinically established; sparse or empty cells identify questions for which the current literature cannot support a conclusion.

### 4.5 Limitations and future research

Several limitations of the evidence base shape the conclusions of this scoping review. Most occlusion-pressure studies involved healthy young adults, with relatively little direct evidence in older adults, clinical populations, or other groups in whom vascular and morphological characteristics may differ. Samples were often recruited by convenience and were modest in size. Confounder selection and adjustment were inconsistent, repeated measurements were not handled uniformly, and the same samples were commonly used to develop and evaluate prediction models. These features make apparently strong model fit within an individual study less informative about performance in a new population. The absence of external validation is particularly important because a useful individualized pressure model must perform beyond the cohort and measurement system in which it was created.

The evidence on downstream responses was much thinner. Two small randomized studies contributed exploratory participant-level analyses, but neither was designed or powered to test effect modification. Allocation concealment and blinding were incompletely reported in these trials, and the relevant baseline-to-change analyses were secondary. Accordingly, the review cannot establish whether any participant characteristic modifies BFR efficacy or safety. Future intervention studies should prespecify a small number of biologically and clinically plausible modifiers, recruit samples large enough for interaction testing, and distinguish prognostic associations from treatment-effect modification. Safety research requires similarly explicit designs, with systematic adverse-event definitions and prospective analyses of potential participant-level susceptibility factors rather than inference from isolated cases.

Future work on AOP/LOP prediction would benefit from a more staged approach. Candidate determinants should be measured with standardized procedures, repeated-observation structures should be modeled appropriately, and model development should report how variables were selected and how overfitting was addressed. Internal validation should be followed by external validation across independent samples, devices, cuff widths, and relevant clinical populations before an equation is used as a substitute for direct measurement. Studies should also keep complete-occlusion outcomes distinct from partial-flow restriction endpoints and report enough procedural detail to determine whether models can reasonably be compared. Age, race or ethnicity, and more detailed body-composition and vascular measures remain underexplored, while sex-related research should test whether any observed difference persists after accounting for limb size and hemodynamics rather than assuming a direct sex effect.

The review process also has limitations. Searches were conducted in PubMed/MEDLINE, Scopus, and Web of Science and were supplemented by complementary searching, but studies indexed exclusively elsewhere may have been missed. The evidence reflects literature available through July 30, 2025. Consistent with the scoping-review objective, the synthesis mapped the nature and distribution of the evidence rather than producing pooled effect estimates or certainty-of-effect ratings. The eligibility threshold for an individual-characteristic analysis was operationally clarified during the review and documented as an amendment to the registered protocol; the full-text set was then reassessed using the same criterion. Critical appraisal was used to qualify interpretation rather than to exclude studies, which preserves the breadth expected of a scoping review while making clear where methodological limitations reduce confidence in the mapped findings.

### 4.6 Practical implications

For practice and future protocol development, the findings support a cautious distinction between measuring occlusion pressure and predicting it. Limb circumference and blood-pressure measures are plausible and repeatedly observed contributors to AOP/LOP, but their effects vary with the measurement setup and none of the published prediction equations was externally validated in the evidence mapped here. When direct individualized AOP/LOP measurement is feasible, it avoids assuming that an equation derived with a different cuff, device, limb, or body position will transfer accurately. Where prediction approaches are used, they should be applied within the methodological context in which they were developed and should not be treated as interchangeable across BFR systems.

Standardization is equally important for repeated measurements within a person. Cuff characteristics, anatomical placement, body position, device, and inflation procedure should be kept consistent when AOP/LOP is reassessed, because changes in these conditions can alter the measured pressure independently of a true change in the participant. The findings also argue against using sex alone as a pressure-prescription rule. Sex-related differences were inconsistent and were often attenuated when limb size or hemodynamic factors were considered. At present, the most defensible role of these participant characteristics is to inform measurement and research design rather than to serve as stand-alone clinical decision thresholds.

The review provides much less guidance for individualizing the expected efficacy or safety of BFR after pressure has been set. Baseline functional or morphological characteristics have not been studied sufficiently to identify reliable responder profiles, and there is no quantitative evidence on participant-level predictors of adverse events within the eligible literature. Clinical decisions about suitability for BFR therefore should not be inferred from the present review as though it established treatment-effect modifiers. Similarly, the absence of eligible studies in populations such as people with vascular malformations should be interpreted as an unanswered safety question, not as evidence that such conditions are either safe indications or contraindications based on this review.

## 5 Conclusion

The available literature is concentrated on participant-level determinants of occlusion pressure rather than on individual variability in the subsequent response to BFR. Limb circumference and blood-pressure measures are the most consistently studied candidate determinants, but their relative contribution varies with cuff characteristics, body position, device, limb, and the specific occlusion-pressure construct. Findings for sex and body composition are less consistent, while age, race or ethnicity, downstream treatment-response modifiers, and safety predictors remain poorly studied. No pressure-prediction equation identified in the review had undergone external validation, and only two studies explored baseline characteristics in relation to later BFR responses without formally testing treatment-effect modification. These findings support standardized individualized measurement of occlusion pressure and further validation of candidate predictors, but they do not justify definitive claims about which participant characteristics modify BFR efficacy or safety.
