# Supplementary material for: Individual factors associated with arterial and limb occlusion pressure in blood flow restriction: a scoping review of predictors and measurement methods
Source: Front Physiol. 2026 Sep 15;17:1958680. doi: 10.3389/fphys.2026.1958680 (PMC13621583; doi:10.3389/fphys.2026.1958680)
Supplement: Supplementary file 1 [file Table1.docx]

**Supplementary material 3.** Summary of the included studies results that analyzed predictors.

| **Study** | **Statistical Test** | **Predictor** | **Dependent Variable** | **SEE** | **R²** | **β / Correlation** | **p** | **Findings** |
| --- | --- | --- | --- | --- | --- | --- | --- | --- |
| ^28^ | Hierarchical linear regression | Arm circ | AOP | NR | 0.612 | β=0.782 | <0.001 | - Arm circ explained 61.2% of AOP variance |
|  |  | bSBP | AOP | NR | 0.689 | β=0.312 | <0.001 | - Adding bSBP increased AOP variance to 68.9% |
| Borges et al.. | Hierarchical linear regression | Arm circ | AOP | NR | 0.370 | β=0.317 | 0.007 | - Arm circ with SBP explained 37% of AOP variance |
|  |  | SBP | AOP | NR | 0.370 | β=0.403 | 0.001 | - SBP with arm circ explained 37% of AOP variance |
|  |  | Sex | AOP | NR | 0.420 | β=0.250 | 0.037 | - Adding sex increased AOP variance to 42% |
| ^27^. | Multiple linear regression | Sex | AOP | NR | Arms: 0.77–0.91, Legs: 0.61–0.87 | Upper (small): β=-1.492, Upper (medium): β=5.319, Lower (medium): β=-17.200, Lower (large): β=-11.935 | ≤0.887 | - Sex significant for leg AOP, less for arms |
|  |  | SBP | AOP | NR | Arms: 0.77–0.91, Legs: 0.61–0.87 | Upper (small): β=1.200, Upper (medium): β=1.284, Lower (medium): β=1.919, Lower (large): β=1.871 | ≤0.058 | - SBP key predictor across limbs and cuff sizes |
|  |  | Seg girth | AOP | NR | Arms: 0.77–0.91, Legs: 0.61–0.87 | Upper (small): β=0.051 (arm), Lower (medium): β=4.178 (mid thigh), Lower (large): β=4.933 (mid thigh) | ≤0.988 | - Mid thigh girth significant for leg AOP |
| ^21^ | Linear mixed model | Thigh circ | LOP | NR | 0.820 | β=0.820 | 0.040 | - Thigh circ significantly predicted LOP |
|  |  | DBP | LOP | NR | NR | β=-0.370 | 0.169 | - DBP not significant for LOP |
|  |  | SBP | LOP | NR | NR | β=0.390 | 0.068 | - SBP not significant for LOP |
|  |  | Sex | LOP | NR | NR | β=2.400 | 0.645 | - Sex not significant for LOP |
| ^30^ | Stepwise multiple regression | MAP | Popliteal AOP | 9.06 | 0.67 | β=0.52 | 0.001 | - MAP significantly predicted popliteal AOP |
|  |  | BMI | Popliteal AOP | 9.06 | 0.67 | β=0.44 | 0.007 | - BMI significantly predicted popliteal AOP |
|  |  | DBP | Popliteal AOP | 8.83 | 0.69 | β=0.53 | 0.001 | - DBP significantly predicted popliteal AOP |
|  |  | Thigh circ | Popliteal AOP | 9.31 | 0.64 | β=0.30 | 0.012 | - Thigh circ significantly predicted popliteal AOP |
|  |  | Arm AT | Brachial AOP | 7.56 | 0.55 | β=-0.39 | 0.018 | - Arm AT significantly predicted brachial AOP |
|  |  | DBP | Brachial AOP | 7.56 | 0.55 | β=0.32 | 0.018 | - DBP significantly predicted brachial AOP |
| ^10^ | Hierarchical linear regression | Arm circ | Brachial AOP | 5 cm: 11.8, 10 cm: 9.1, 12 cm: 8.6 | 5 cm: 0.651, 10 cm: 0.570, 12 cm: 0.557 | β=0.715 (5 cm), 0.541 (10 cm), 0.524 (12 cm) | <0.001 | - Arm circ strongest predictor across cuff sizes |
|  |  | bSBP | Brachial AOP | 5 cm: 11.8, 10 cm: 9.1, 12 cm: 8.6 | 5 cm: 0.651, 10 cm: 0.570, 12 cm: 0.557 | β=0.521 (5 cm), 0.537 (10 cm), 0.568 (12 cm) | <0.001 | - bSBP key predictor across cuff sizes |
|  |  | Sex | Brachial AOP | 5 cm: 11.8, 10 cm: 9.1, 12 cm: 8.6 | 5 cm: 0.651, 10 cm: 0.570, 12 cm: 0.557 | β=0.073 (5 cm), 0.227 (10 cm), 0.246 (12 cm) | ≤0.315 | - Sex significant for 10 cm, 12 cm cuffs |
| ^35^ | Linear regression | Arm circ | AOP | Supine: 18.24, Seated: 15.29, Standing: 16.61 | Supine: 0.398, Seated: 0.514, Standing: 0.451 | r=0.598 (supine), 0.717 (seated), 0.671 (standing) | <0.001 | - Arm circ explained 39.8–51.4% of AOP variance |
|  |  | SBP | AOP | Supine: 18.39, Seated: 18.07, Standing: 19.58 | Supine: 0.348, Seated: 0.273, Standing: 0.237 | r=0.590 (supine), 0.522 (seated), 0.486 (standing) | ≤0.001 | - SBP explained 23.7–34.8% of AOP variance |
| (Loenneke et al., 2012) | Hierarchical linear regression | Thigh circ | AOP | Wide: 15.884, Narrow: 35.611 | Wide: 0.632, Narrow: 0.297 | β=0.587 (wide), 0.375 (narrow) | <0.017 | - Thigh circ strongest predictor for both cuffs |
|  |  | ABP | AOP | Wide: 15.884 | Wide: 0.632 | β=0.244 (wide) | <0.005 | - ABP significant for wide cuffs |
|  |  | DBP | AOP | Wide: 15.884, Narrow: 35.611 | Wide: 0.632, Narrow: 0.297 | β=0.216 (wide), 0.265 (narrow) | <0.017 | - DBP significant for both cuffs |
|  |  | fCSA | AOP | Narrow: 35.611 | Narrow: 0.297 | β=0.467 (narrow) | <0.017 | - fCSA significant for narrow cuffs |
| (Loenneke et al., 2015) | Hierarchical linear regression | Arm circ | Upper body AOP | 9.5 | 0.439 | β=0.419 | <0.001 | - Arm circ key predictor for upper AOP |
|  |  | SBP | Upper body AOP | 9.5 | 0.439 | β=0.394 | <0.001 | - SBP key predictor for upper AOP |
|  |  | Thigh circ | Lower body AOP | 23.5 | 0.492 | β=0.579 | <0.001 | - Thigh circ strongest for lower AOP |
|  |  | SBP | Lower body AOP | 23.5 | 0.492 | β=0.281 | <0.001 | - SBP significant for lower AOP |
| ^34^ | Hierarchical linear regression | SBP | LL-AOP | Large: Supine 11.248, Seated 17.012, Standing 15.104 | Large: Supine 0.395, Seated 0.363, Standing 0.540 | β=0.456 (supine), 0.388 (seated), 0.489 (standing) | ≤0.084 | - SBP key for large cuff across positions |
|  |  | Thigh circ | LL-AOP | Large: Supine 11.248, Seated 17.012, Standing 15.104; Medium: Supine 18.089, Seated 15.886, Standing 17.491 | Large: Supine 0.395, Seated 0.363, Standing 0.540; Medium: Supine 0.475, Seated 0.614, Standing 0.541 | β=0.253 (supine, large), 0.327 (seated, large), 0.428 (standing, large), 0.519 (supine, medium), 0.739 (seated, medium), 0.611 (standing, medium) | ≤0.249 | - Thigh circ key for medium cuff, less for large |
|  |  | DBP | LL-AOP | Medium: Supine 18.089 | Medium: Supine 0.475 | β=0.325 (supine, medium) | ≤0.249 | - DBP significant for medium cuff in supine |
| ^15^ | Multiple linear regression | Thigh circ | Lower body AOP | Wide: Supine 15.935, Seated 15.844; Narrow: Supine 71.529, Seated 86.652 | Wide: Supine 0.561, Seated 0.659; Narrow: Supine 0.302, Seated 0.340 | β=0.525 (supine, wide), 0.572 (seated, wide), 0.604 (supine, narrow), 0.629 (seated, narrow) | <0.01 | - Thigh circ strongest predictor for both cuffs |
|  |  | DBP | Lower body AOP | Wide: Supine 15.935, Seated 15.844 | Wide: Supine 0.561, Seated 0.659 | β=0.379 (supine, wide), 0.318 (seated, wide) | <0.05 | - DBP adds variance for wide cuff |
| ^17^ | Linear regression | Leg circ | AOP | NR | NR | β=3.23 | ≤0.009 | - Leg circ sole AOP predictor |
| ^33^ | Linear regression | Thigh circ | AOP | NR | NR | β=2.43 | 0.0278 | - Thigh circ only significant AOP predictor |
| ^20^. | Pearson correlation | LL muscle mass | AOP | NR | 0.433 | r²=0.433, β=-0.004 | ≤0.027 | - Negative correlation with AOP |
|  |  | LL bone mass | AOP | NR | 0.356 | r²=0.356, β=-0.047 | ≤0.027 | - Negative correlation with AOP |
|  |  | LL fat mass | AOP | NR | 0.384 | r²=0.384, β=0.005 | ≤0.027 | - Positive correlation with AOP |
|  |  | LL circ | AOP | NR | 0.316 | r²=0.316, β=0.050 | ≤0.027 | - Positive correlation with AOP |

AOP: Arterial Occlusion Pressure; LL-AOP: Lower Limb Arterial Occlusion Pressure; LOP: Limb Occlusion Pressure; BFR: Blood Flow Restriction; circ: Circumference; SBP: Systolic Blood Pressure; bSBP: Brachial Systolic Blood Pressure; DBP: Diastolic Blood Pressure; bDBP: Brachial Diastolic Blood Pressure; MAP: Mean Arterial Pressure; ABP: Ankle Blood Pressure; mCSA: Muscle Cross-Sectional Area; fCSA: Fat Cross-Sectional Area; MTH: Muscle Thickness; FTH: Fat Thickness; AT: Adipose Tissue; seg: Segment; NR: Not Reported; LL: Lower limb
