# Supplementary material for: Individual factors associated with arterial and limb occlusion pressure in blood flow restriction: a scoping review of predictors and measurement methods
Source: Front Physiol. 2026 Sep 15;17:1958680. doi: 10.3389/fphys.2026.1958680 (PMC13621583; doi:10.3389/fphys.2026.1958680)
Supplement: Supplementary file 2 [file Table2.docx]

Supplementary material 4. Summary of the included studies results that did not analyze predictors.

| **Study** | **Statistical Test** | **Intervention/groups** | **Dependent Variable** | **Effect Size (ES)** | **Comparison/interaction** | **p** | **Efficacy of AOP/BFRT** | **Safety of AOP/BFRT** |
| --- | --- | --- | --- | --- | --- | --- | --- | --- |
| (Barbalho et al., 2019) | Repeated-measures ANOVA | Passive mobilization with BFR vs. control (no BFR) on lower limbs | Thigh circumference (cm) | Control: 1.40;  BFR: 0.86 | Within-subjects: F = 257.81, η² = 0.87; Between-subjects: F = 0.23, η² = 0.01; | Within-subjects: <0.001;  Between-subjects: 0.63; | BFR limb showed reduced muscle atrophy compared to control (–2.1 vs. –2.8 mm in muscle thickness, P=0.001; –2.5 vs. –3.6 cm in thigh circumference, P=0.001); 6.5% lower muscle wasting rate with BFR | No serious adverse effects reported; BFR well-tolerated in ICU coma patients |
|  |  |  | Quadriceps muscle thickness (mm) | Control: 1.03;  BFR: 0.80 | Within-subjects: F = 334.6, η² = 0.90; Between-subjects: F = 0.22, η² = 0.01; | Within: <0.001; Between: 0.64; |  |  |
| ^49^ | Friedman test with post hoc analysis | AOP measurements in supine, seated, and standing positions on dominant lower limb | AOP (mmHg) | NR | χ²(2) = 90.04 | <0.001 | AOP varied by position: lower in supine (187.00 ± 32.5 mmHg) vs. seated (204.00 ± 28.5 mmHg, p<0.001) and standing (241.50 ± 49.3 mmHg, p<0.001); seated lower than standing (p<0.001); accurate position-specific AOP measurement enhances BFR efficacy | No adverse events reported during AOP measurements in any position |
| ^44^ | Two-way ANOVA | Blood pressure measurements with different cuff widths (5 cm, 10 cm, 12 cm) in right arm | Systolic blood pressure (mmHg) | NR | NR | <0.001 | Inverse relationship between cuff width and systolic pressure for both men and women.  Men had higher pressures than women (p < 0.001).  - Significant differences across all cuff widths (5 cm > 10 cm > 12 cm, p < 0.001) | NR |
|  |  |  | Diastolic blood pressure (mmHg) | NR | NR | <0.001 | Inverse relationship between cuff width and diastolic pressure for both men and women.  Men had higher pressures than women (p < 0.001).  Significant differences across all cuff widths (5 cm > 10 cm > 12 cm, p < 0.001) |  |
|  |  |  | Brachial artery blood flow (mL/min) | NR | Main effects: cuff (p = 0.002), pressure (p < 0.0001); No cuff by pressure interaction (p = 0.272) | Cuff: 0.002; Pressure: <0.0001 | - Significant differences: 5 cm vs. 10 cm (p < 0.01); 10 cm vs. 12 cm at rest and 10–30% pressures.  - Blood flow stable above 40% occlusion pressure |  |
|  |  |  | Peak systolic velocity (VPEAK, cm/s) | NR | Cuff by pressure interaction (p < 0.0001) | <0.0001 | - Minimal VPEAK decreases until higher pressures; greater declines with 5 cm cuff at higher pressures.  - Significant differences at 70–100% occlusion: 5 cm vs. 10 cm/12 cm (p < 0.01).  - VPEAK stable across most pressures for 10 cm and 12 cm cuffs |  |
|  |  |  | Shear rate (s⁻¹) | NR | Main effects: cuff (p = 0.0021), pressure (p < 0.00005); No cuff by pressure interaction (p = 0.487) | Cuff: 0.0021; Pressure: <0.000 | - Shear rate decreased with increasing pressure, tracking blood flow reduction. - Significant differences: 10 cm vs. 12 cm (p < 0.01); rest vs. 20–100% pressures (p < 0.001) |  |
| ^22^ | ANOVA | BFR point determination on upper and lower limbs (right and left) in supine, seated, and standing positions | BFR point (mmHg) | NR | NR | Position: ≤0.041; Sex: <0.001; Limb: ≤0.019; Segment: ≤0.014 | - Higher BFR points in sitting/standing vs. lying for upper (p≤0.041) and lower limbs (p<0.001); males > females (p<0.001); lower > upper limbs (p≤0.019); left > right segment in lying (p≤0.014) | NR |
| ^46^ | ANOVA | BFR therapy with hand therapy vs. hand therapy alone (control) | Pain with activity | NR | BFR vs. control (magnitude of change, 0–8 weeks) | p = 0.03 | - Greater pain reduction in BFR vs. control over 8 weeks | Well-tolerated, no complications, no deleterious effects on radiographic outcomes |
|  |  |  | PRWE scores | NR |  | p = 0.01 | - Greater PRWE score reduction in BFR vs. control over 8 weeks |  |
|  |  |  | Wrist/forearm ROM, DASH scores, grip strength, pinch strength, radiographic parameters | NR | BFR vs. control (all time points and magnitude of change, 0–8 weeks) | ≥0.07 | - No significant differences in ROM, DASH, grip/pinch strength, or radiographic parameters between groups |  |
| ^43^ | Two-way ANOVA | BFR-RT at 30% 1RM vs. HL-RT at 70% 1RM on injured/non-injured limbs | Session knee pain | d=2.5 (injured) | Group x time: F(14,252)=2.174, d=0.1; Group: F(14,252)=57.898, d=0.8; Time: F(14,252)=15.667, d=0.5 | Group x time: <0.05; Group/Time: <0.01 | - Lower session knee pain in BFR-RT vs. HL-RT (all sessions, p<0.05) | No adverse events reported; BFR-RT well-tolerated with high adherence despite increased muscle pain |
|  |  |  | 24h post-training knee pain | d=3.1 (injured) | Group x time: F(3.519,77.413)=26.880, d=0.9; Group: F(3.519,77.413)=34.959, d=0.9; Time: F(3.519,77.413)=131.628, d=1.0 | Group x time/Group/Time: <0.01 | - Lower 24h post-training knee pain in BFR-RT vs. HL-RT (all timepoints, p<0.01); greater pain reduction in BFR-RT (sessions 4–10, p<0.01) |  |
|  |  |  | Muscle pain | d=0.8 (injured), d=0.3 (non-injured) | Injured: Group x time: F(14,154)=4.047, d=0.2; Group: F(1,11)=5.091, d=0.8; Time: F(14,154)=17.417, d=0.6; Non-injured: Group x time: F(14,126)=2.701, d=0.2; Group: F(14,126)=4.790, d=0.3; Time: F(14,126)=2.217, d=0.4 | Group x time: <0.01; Group/Time: <0.05 | - Higher muscle pain in BFR-RT vs. HL-RT (all sessions, p<0.05) |  |
| ^47^ | Two-way ANOVA | KAATSU RT vs. control post-cardiac surgery | Body mass (kg) | Control: d=0.78–0.88; KAATSU: d=0.24–1.35 | Group x time: F=4.8 | 0.016 | - KAATSU RT: body mass decreased early post-surgery (p=0.003) but returned to baseline after 3 months; control: no change | No side effects, no hospitalizations, no cardiac symptom exacerbation, no DOMS, no subcutaneous hemorrhage or petechiae; CPK normal, D-dimer normalized post-3 months |
|  |  |  | BMI (kg/m²), body fat volume (kg) | NR | Time main effect: BMI F=4.7, body fat volume F=4.6 | BMI: 0.015  Body fat: 0.016 | - No group differences; BMI and body fat volume changed over time (p<0.05) |  |
|  |  |  | Muscle thickness (MTH, cm), skeletal muscle mass index (SMI, kg/m²) | Control: d=0.01–0.88; KAATSU: d=0.92–1.85 | Group x time: MTH F=12.0, SMI F=3.8 | MTH: 0.0001; SMI: 0.03 | - KAATSU RT: MTH/SMI decreased early post-surgery (p≤0.04) but increased above baseline after 3 months (p≤0.02); control: no change |  |
|  |  |  | Walking speed (m/s) | Control: d=0.14–0.41; KAATSU: d=0.54–1.9 | Group x time: F=5.1 | 0.01 | - KAATSU RT: walking speed increased above baseline after 3 months (p=0.001); control: no change |  |
|  |  |  | Handgrip strength (kgf) | NR | Time main effect: F=5.7 | 0.007 | - No group differences; handgrip strength changed over time (p<0.01) |  |
|  |  |  | Knee extensor strength (kgf) | Control: d=0.45–0.70; KAATSU: d=0.16–1.53 | Group x time: F=7.4 | 0.002 | - KAATSU RT: knee extensor strength increased above baseline after 3 months (p=0.0002); control: no change |  |
| ^48^ | Paired t-test | 12-week BFR training program (pre vs. post) | Muscle strength (1RM leg press, knee extension) | Leg press: d=0.42; Knee extension: d=0.87 | Pre vs. post intervention | <0.001 | - Increased 1RM leg press (19.6%) and knee extension (25.2%) post-BFR training | No adverse events, no excessive fatigue, pain, or muscle soreness; CK and aldolase unchanged (p>0.05) |
|  |  |  | Physical function (TST, TUG scores) | TST: d=0.87; TUG: d=-0.29 |  | TST: <0.001; TUG: 0.002 | - Improved TST (15.1%) and TUG (-4.5%) scores post-BFR training |  |
|  |  |  | Quadriceps CSA (cm²) | d=0.21 |  | 0.01 | - Increased quadriceps CSA (4.57%) post-BFR training |  |
|  |  |  | Quality of life (SF-36, HAQ, VAS) | SF-36: d=0.65–2.52; HAQ: d=-0.49; VAS patient: d=-1.51; VAS physician: d=-1.21 |  | 0.002 | - Improved SF-36 (all components), HAQ, and VAS (patient/physician) scores post-BFR training |  |
|  |  |  | CK, aldolase | NR |  | ≥0.405 | - No changes in CK or aldolase post-BFR training |  |
| ^42^ | one-way ANOVA | KAATSU (50–200 mmHg) in seated/standing (pre-bed rest) and post-24h bed rest | Heart rate (HR, bpm) | NR | Pre vs. KAATSU (100–200 mmHg); Pre vs. standing; Post-bed rest: Pre vs. KAATSU (50–100 mmHg), Pre vs. standing | ≤0.01 | - Increased HR with KAATSU (100–200 mmHg, seated pre-bed rest; 50–100 mmHg, post-bed rest) and standing | One subject developed presyncope due to hypotension during 100 mmHg KAATSU post-bed rest; no other symptoms reported in remaining subjects |
|  |  |  | Stroke volume (SV, ml) |  |  | ≤0.01 | - Decreased SV with KAATSU (100–200 mmHg, seated pre-bed rest; 50–100 mmHg, post-bed rest) and standing |  |
|  |  |  | Cardiac output (CO, l/min) |  | Pre vs. KAATSU (50–100 mmHg, post-bed rest) | ≤0.01 | - Decreased CO with KAATSU (50–100 mmHg, post-bed rest, and no change pre-bed rest |  |
|  |  |  | Total peripheral resistance (TPR, dyne s cm⁻⁵) |  | Pre vs. KAATSU (50–100 mmHg, post-bed rest); Pre vs. standing | ≤0.05 | - Increased TPR with KAATSU (50–100 mmHg, post-bed rest) and standing |  |
|  |  |  | Inferior vena cava diameter (IVC, cm), left ventricular diastolic diameter (LVDd, mm) |  | Pre vs. post 24h bed rest; KAATSU (50–100 mmHg, post-bed rest) | ≤0.05 | - Decreased IVC after 24h bed rest and with KAATSU (50–100 mmHg, post-bed rest, decreased LVDd with KAATSU (50–100 mmHg, post-bed rest |  |
|  |  |  | Blood pressure (sBP, mBP, dBP, mmHg) |  | Pre vs. standing; Post-bed rest: Pre vs. KAATSU (50–100 mmHg) | ≤0.05 | - Increased mBP with standing (p<0.05); no change with KAATSU |  |
|  |  |  | Hemostatic markers (NOR, PRA, ADH) |  | Pre vs. KAATSU (50–100 mmHg, post-bed rest) | ≤0.05 | - Increased NOR (50–100 mmHg), PRA, ADH (100 mmHg) with KAATSU post-bed rest |  |
|  |  |  | Body mass (kg), blood/plasma volume (BV/PV, %), Hct (%), Hb (mg/dl) |  | Pre vs. post 24h bed rest | <0.01 | - Decreased body mass, BV, PV; increased Hct, Hb after 24h bed rest |  |

BFR: Blood Flow Restriction; KAATSU: A specific type of BFR training; ANOVA: Analysis of Variance; AOP: Arterial Occlusion Pressure; 1RM: One Repetition Maximum; PRWE: Patient-Rated Wrist Evaluation; DASH: Disabilities of the Arm, Shoulder, and Hand; ROM: Range of Motion; HL-RT: High-Load Resistance Training; MTH: Muscle Thickness; SMI: Skeletal Muscle Mass Index; BMI: Body Mass Index; TST: Timed Stands Test; TUG: Timed Up and Go; CSA: Cross-Sectional Area; SF-36: 36-Item Short Form Survey; HAQ: Health Assessment Questionnaire; VAS: Visual Analog Scale; CK: Creatine Kinase; HR: Heart Rate; SV: Stroke Volume; CO: Cardiac Output; TPR: Total Peripheral Resistance; IVC: Inferior Vena Cava; LVDd: Left Ventricular Diastolic Diameter; sBP: Systolic Blood Pressure; mBP: Mean Blood Pressure; dBP: Diastolic Blood Pressure; NOR: Norepinephrine; PRA: Plasma Renin Activity; ADH: Antidiuretic Hormone; BV: Blood Volume; PV: Plasma Volume; Hct: Hematocrit; Hb: Hemoglobin; VPEAK: Peak Systolic Velocity
