# Supplementary material for: Individual factors associated with arterial and limb occlusion pressure in blood flow restriction: a scoping review of predictors and measurement methods
Source: Front Physiol. 2026 Sep 15;17:1958680. doi: 10.3389/fphys.2026.1958680 (PMC13621583; doi:10.3389/fphys.2026.1958680)
Supplement: Supplementary file 3 [file Table3.docx]

**Supplementary material 5.** Summary of the case report studies results.

| **Study** | **Intervention/groups** | **Dependent Variable** | **Comparison/interaction** | **Efficacy of AOP/BFRT** | **Safety of AOP/BFRT** |
| --- | --- | --- | --- | --- | --- |
| ^36^ | Single BFR exercise session (bilateral knee-extension, elbow-flexion at 25% intensity, 167 mmHg cuff pressure) | Serum creatine kinase (CK, IU/L), pain, ambulation difficulty | Post-BFR exercise (48h, hospitalization, discharge) | - Post-BFR: thigh pain (7.5/10), ambulation difficulty, elevated serum CK (18,022 IU/L at 48h, peak 36,000 IU/L, 6,000 IU/L at discharge); diagnosed exertional rhabdomyolysis | NR |
| ^37^ | 12-week BFR/LIRT program (pre vs. 6wk vs. 12wk) | MSWS-12, FSS, PSFS (running, stairs, driving) | Baseline vs. 6wk vs. 12wk | - MSWS-12: improved from 52 to 31.6 (6wk) and 33 (12wk); FSS: unchanged at 6wk (4.0), improved to 2.2 (12wk); PSFS: running (0 to 6), stairs (2 to 10), driving (3 to 9) improved, exceeding MDC | Well-tolerated, no adverse events, high adherence (21/24 sessions) despite increased exertion with higher intensity and AOP (40–80%) |
|  |  | Muscle strength (hip, knee, ankle, kg) |  | - Strength improved at 12wk (8/12 measures exceeded MDC); 2/14 exceeded MDC at 6wk |  |
| ^38^ | Single BFR low-load knee extension session (12 kg, 100 mmHg cuff pressure) | Serum creatine kinase (CK, U/L), aspartate aminotransferase (AST, U/L), muscle soreness | Post-BFR exercise (48h, 3–5 days, 18 days) | - Post-BFR: extreme quadriceps soreness, CK elevated to 12,400 U/L (48h), 12,950 U/L (3 days), 6,300 U/L (5 days); AST 440 to 255 U/L; diagnosed rhabdomyolysis; returned to training after 18 days | A single BFR low-load knee extension session (12 kg, 100 mmHg cuff pressure) caused exertional rhabdomyolysis with extreme quadriceps soreness, elevated CK and AST. Required hospitalization |
| ^39^ | 12-week LL-BFRT (first 12 sessions) + HL-ST (next 12 sessions) | Knee extensor/flexor strength (Nm), 1-RM leg press/extension (kg) | Pre vs. post intervention | - Knee extensor strength: right 18.3 to 113.6 Nm (521%), left 20.4 to 108.8 Nm (433%); knee flexor strength: right 35.4 to 69.2 Nm (95%), left 30.4 to 86.4 Nm (184%); 1-RM leg press: 45.4 to 82.5 kg (82%), leg extension: 5.1 to 17.5 kg (243%) | Well-tolerated, no adverse events (e.g., muscle soreness, vascular issues, joint pain); high adherence to 24 sessions |
|  |  | 6MWT (m), 1MSTS (repetitions) |  | - 6MWT: 468 to 530 m (11%); 1MSTS: 13 to 27 repetitions (108%) |  |
|  |  | CAT score (points) |  | - CAT score: 15 to 9 points (40% reduction) |  |
|  |  | Lung function (FEV1, FVC, TLCO) |  | - No relevant changes in FEV1 (1.52 to 1.58 L), FVC (2.57 to 2.51 L), TLCO (4.6 to 4.3 mmol/kPa/min) |  |
| ^40^ | KAATSU training (30 min–1 hr, 3x/week, constant pressure) | Left upper limb symptoms, D-dimer (μg/mL), imaging (CT, ultrasonography, scintigraphy) | Post-KAATSU training (6 days, hospital course, 1-year follow-up) | NR | KAATSU training associated with left subclavian vein thrombosis and pulmonary thromboembolism; elevated D-dimer (1.7 μg/mL), thrombus detected, persistent symptoms on elevation; patient quit KAATSU post-discharge, no symptom relapse after 3 years |
| ^41^ | Single BFR training session (squats, 3 sets of 20 reps) | Serum CPK (U/L), urine myoglobin (ng/mL), muscle pain, fever, CRP (mg/dL), AST/ALT/LDH (U/L) | Post-BFR (Day 1–18) | NR | BFR training associated with acute rhabdomyolysis; severe muscle pain, elevated CPK (56,475 U/L at admission, peak 89,824 U/L), urine myoglobin (>3000 ng/mL), and elevated muscle enzymes (AST, ALT, LDH); required hospitalization, resolved with IV fluids and antibiotics |

BFR: Blood Flow Restriction; LIRT: Low-Intensity Resistance Training; LL-BFRT: Low-Load Blood Flow Restriction Training; HL-ST: High-Load Strength Training; KAATSU: A specific type of BFR training; MSWS-12: 12-Item Multiple Sclerosis Walking Scale; FSS: Fatigue Severity Scale; PSFS: Patient-Specific Functional Scale; MDC: Minimum Detectable Change; CK: Creatine Kinase; AST: Aspartate Aminotransferase; 1-RM: One Repetition Maximum; 6MWT: 6-Minute Walk Test; 1MSTS: 1-Minute Sit-to-Stand Test; CAT: COPD Assessment Test; FEV1: Forced Expiratory Volume in 1 Second; FVC: Forced Vital Capacity; TLCO: Lung Diffusion Capacity of Carbon Monoxide; CPK: Creatine Phosphokinase; CRP: C-Reactive Protein; ALT: Alanine Aminotransferase; LDH: Lactic Dehydrogenase
