# Supplementary material for: Individual factors associated with arterial and limb occlusion pressure in blood flow restriction: a scoping review of predictors and measurement methods
Source: Front Physiol. 2026 Sep 15;17:1958680. doi: 10.3389/fphys.2026.1958680 (PMC13621583; doi:10.3389/fphys.2026.1958680)
Supplement: Supplementary file 4 [file Table4.docx]

Supplementary Material S1. Full-text reports excluded after eligibility assessment

| **Primary exclusion cluster** | **Report** | **Primary reason for exclusion** |
| --- | --- | --- |
| Non-primary publication | McEwen et al. (2019) | Narrative/review paper; not eligible primary human research. |
| Non-primary publication | de Queiros et al. (2024) | Scoping review and recommendations paper; not eligible primary human research. |
| Methodological/environmental only | Hughes et al. (2018) | Examined body position and reliability of lower-limb AOP without evaluating an eligible participant-level predictor, moderator, or baseline correlate. |
| Methodological/environmental only | Mouser et al. (2017) | Examined cuff-specific hemodynamic responses rather than participant-level characteristics associated with eligible individualized outcomes. |
| Methodological/environmental only | Nakajima et al. (2008) | Examined KAATSU during simulated weightlessness without an eligible participant-characteristic analysis. |
| Methodological/environmental only | Swain and Caplan (2024) | Examined body tilting and reduced-gravity analogues as procedural conditions rather than participant-level characteristics. |
| Efficacy/tolerability only | Cancio et al. (2019) | Rehabilitation study reporting efficacy and safety outcomes, but without participant-level predictor, moderator, or baseline-response analysis. |
| Efficacy/tolerability only | Hughes et al. (2019) | Randomized rehabilitation trial focused on comfort and pain; participant characteristics were not analyzed as determinants of response. |
| Efficacy/tolerability only | Mattar et al. (2014) | Pre–post clinical study reporting efficacy and safety only; participant characteristics were not analyzed in relation to response. |
| Efficacy/tolerability only | Scott et al. (2017) | Training-intervention study in athletes without eligible analysis of participant-level heterogeneity. |
| Case report | Clark and Manini (2016) | Single adverse-event case report without analyzable participant-characteristic relationships. |
| Case report | Cohen et al. (2021) | Single clinical case report without analyzable participant-characteristic relationships. |
| Case report | Iversen and Røstad (2010) | Single adverse-event case report without analyzable participant-characteristic relationships. |
| Case report | Kohlbrenner et al. (2021) | Single clinical case report without analyzable participant-characteristic relationships. |
| Case report | Noto et al. (2017) | Single thrombotic-event case report without analyzable participant-characteristic relationships. |
| Case report | Tabata et al. (2016) | Single adverse-event case report without analyzable participant-characteristic relationships. |

**References**

Cancio, J. M., Sgromolo, N. M., & Rhee, P. C. (2019). Blood flow restriction therapy after closed treatment of distal radius fractures. Journal of Wrist Surgery, 8, 288–294. https://doi.org/10.1055/s-0039-1685455

Clark, B. C., & Manini, T. M. (2016). Can KAATSU exercise cause rhabdomyolysis? Clinical Journal of Sport Medicine. Advance online publication. https://doi.org/10.1097/JSM.0000000000000309

Cohen, E. T., Cleffi, N., Ingersoll, M., & Karpatkin, H. I. (2021). Blood-flow restriction training for a person with primary progressive multiple sclerosis: A case report. PTJ: Physical Therapy & Rehabilitation Journal, 101, pzaa224. https://doi.org/10.1093/ptj/pzaa224

de Queiros, V. S., Rolnick, N., Wilde, P., de Melo, A., Cabral, B. G. A. T., & Dantas, P. M. S. (2024). Measuring arterial occlusion pressure for training with blood flow restriction: A scoping review and recommendations for measurement. Sport Sciences for Health, 20, 259–272. https://doi.org/10.1007/s11332-023-01135-y

Hughes, L., Jeffries, O., Waldron, M., Rosenblatt, B., Gissane, C., Paton, B., & Patterson, S. D. (2018). Influence and reliability of lower-limb arterial occlusion pressure at different body positions. PeerJ, 6, e4697. https://doi.org/10.7717/peerj.4697

Hughes, L., Patterson, S. D., Haddad, F., Rosenblatt, B., Gissane, C., McCarthy, D., Clarke, T., Ferris, G., Dawes, J., & Paton, B. (2019). Examination of the comfort and pain experienced with blood flow restriction training during post-surgery rehabilitation of anterior cruciate ligament reconstruction patients: A UK National Health Service trial. Physical Therapy in Sport, 39, 90–98. https://doi.org/10.1016/j.ptsp.2019.06.014

Iversen, E., & Røstad, V. (2010). Low-load ischemic exercise–induced rhabdomyolysis. Clinical Journal of Sport Medicine, 20, 218–219. https://doi.org/10.1097/JSM.0b013e3181df8d10

Kohlbrenner, D., Aregger, C., Osswald, M., Sievi, N. A., & Clarenbach, C. F. (2021). Blood-flow–restricted strength training combined with high-load strength and endurance training in pulmonary rehabilitation for COPD: A case report. PTJ: Physical Therapy & Rehabilitation Journal, 101, pzab063. https://doi.org/10.1093/ptj/pzab063

Mattar, M. A., Gualano, B., Perandini, L. A., Shinjo, S. K., Lima, F. R., Sá-Pinto, A. L., & Roschel, H. (2014). Safety and possible effects of low-intensity resistance training associated with partial blood flow restriction in polymyositis and dermatomyositis. Arthritis Research & Therapy, 16, 473. https://doi.org/10.1186/s13075-014-0473-5

McEwen, J. A., Owens, J. G., & Jeyasurya, J. (2019). Why is it crucial to use personalized occlusion pressures in blood flow restriction (BFR) rehabilitation? Journal of Medical and Biological Engineering, 39, 173–177. https://doi.org/10.1007/s40846-018-0397-7

Mouser, J. G., Dankel, S. J., Jessee, M. B., Mattocks, K. T., Buckner, S. L., Counts, B. R., & Loenneke, J. P. (2017). A tale of three cuffs: The hemodynamics of blood flow restriction. European Journal of Applied Physiology, 117, 1493–1499. https://doi.org/10.1007/s00421-017-3644-7

Nakajima, T., Iida, H., Kurano, M., Takano, H., Morita, T., Meguro, K., Sato, Y., Yamazaki, Y., Kawashima, S., Ohshima, H., Tachibana, S., Ishii, N., & Abe, T. (2008). Hemodynamic responses to simulated weightlessness of 24-h head-down bed rest and KAATSU blood flow restriction. European Journal of Applied Physiology, 104, 727–737. https://doi.org/10.1007/s00421-008-0834-3

Noto, T., Hashimoto, G., Takagi, T., Awaya, T., Araki, T., Shiba, M., Iijima, R., Hara, H., Moroi, M., Nakamura, M., & Sugi, K. (2017). Paget–Schroetter syndrome resulting from thoracic outlet syndrome and KAATSU training. Internal Medicine, 56, 2595–2601. https://doi.org/10.2169/internalmedicine.7937-16

Scott, B. R., Peiffer, J. J., & Goods, P. S. R. (2017). The effects of supplementary low-load blood flow restriction training on morphological and performance-based adaptations in team sport athletes. Journal of Strength and Conditioning Research, 31, 2147–2154. https://doi.org/10.1519/JSC.0000000000001671

Swain, P., & Caplan, N. (2024). Blood flow restriction: The acute effects of body tilting and reduced gravity analogues on limb occlusion pressure. Experimental Physiology. Advance online publication. https://doi.org/10.1113/EP091874

Tabata, S., Suzuki, Y., Azuma, K., & Matsumoto, H. (2016). Rhabdomyolysis after performing blood flow restriction training: A case report. Journal of Strength and Conditioning Research, 30, 2064–2068. https://doi.org/10.1519/JSC.0000000000001295
