# Supplementary material for: Individual factors associated with arterial and limb occlusion pressure in blood flow restriction: a scoping review of predictors and measurement methods
Source: Front Physiol. 2026 Sep 15;17:1958680. doi: 10.3389/fphys.2026.1958680 (PMC13621583; doi:10.3389/fphys.2026.1958680)
Supplement: Supplementary file 5 [file Table5.docx]

**Supplementary Material S2. JBI critical appraisal of the included evidence**

Critical appraisal was applied according to the analytical question and study design. The 15 studies in the occlusion-pressure domain were appraised using the JBI Critical Appraisal Checklist for Analytical Cross Sectional Studies, with the appraisal focused on the participant-characteristic association or prediction analysis. Procedural randomization of cuff order, limb, inflation protocol, or body position was treated as a methodological design feature rather than as evidence of an intervention-effect trial. Barbalho et al. (2019) and Ogawa et al. (2021) were appraised using the JBI Critical Appraisal Checklist for Randomized Controlled Trials. No numerical quality score or generic overall risk category was calculated.

**Table S2.1. JBI appraisal of occlusion-pressure association/prediction studies (n = 15).**

| **Study** | **Q1** | **Q2** | **Q3** | **Q4** | **Q5** | **Q6** | **Q7** | **Q8** |
| --- | --- | --- | --- | --- | --- | --- | --- | --- |
| Barnett et al. (2016) | Y | Y | Y | Y | U | Y | Y | Y |
| Borges et al. (2018) | Y | Y | Y | Y | Y | Y | Y | Y |
| Brown et al. (2018) | Y | Y | Y | Y | Y | Y | Y | U |
| Evin et al. (2021) | Y | Y | Y | Y | Y | Y | Y | Y |
| Hunt et al. (2016) | Y | Y | Y | Y | Y | Y | Y | Y |
| Jessee et al. (2016) | Y | Y | Y | Y | Y | Y | Y | Y |
| Karanasios et al. (2022) | Y | Y | Y | Y | Y | Y | Y | Y |
| Loenneke et al. (2012) | Y | Y | Y | Y | Y | Y | U | Y |
| Loenneke et al. (2015) | Y | Y | Y | Y | Y | Y | Y | Y |
| de Queiros et al. (2024) | Y | Y | Y | Y | Y | Y | U | Y |
| Sieljacks et al. (2018) | Y | Y | Y | Y | Y | Y | U | U |
| Tafuna’i et al. (2021) | Y | Y | Y | Y | Y | Y | Y | Y |
| Vehrs et al. (2023) | Y | Y | Y | Y | Y | Y | Y | Y |
| Walden et al. (2024) | Y | Y | Y | Y | Y | Y | Y | Y |
| Rodrigues Neto et al. (2018) | Y | Y | Y | Y | N | N | Y | N |

Judgment options: Y = Yes; N = No; U = Unclear; NA = Not applicable. The JBI items were interpreted in relation to the participant-characteristic association/prediction question: Q3 was applied to measurement of the participant-level characteristic(s); Q4 and Q7 were applied to definition and measurement of the occlusion-pressure outcome; Q5-Q6 addressed identification and handling of relevant covariates/confounding.

| **Item** | **JBI item** |
| --- | --- |
| **Q1** | Were the criteria for inclusion in the sample clearly defined? |
| **Q2** | Were the study subjects and the setting described in detail? |
| **Q3** | Was the exposure measured in a valid and reliable way? |
| **Q4** | Were objective, standard criteria used for measurement of the condition? |
| **Q5** | Were confounding factors identified? |
| **Q6** | Were strategies to deal with confounding factors stated? |
| **Q7** | Were the outcomes measured in a valid and reliable way? |
| **Q8** | Was appropriate statistical analysis used? |

**Table s2.2. JBI appraisal of randomized controlled trials (n = 2).**

| **Study** | **Q1** | **Q2** | **Q3** | **Q4** | **Q5** | **Q6** | **Q7** | **Q8** | **Q9** | **Q10** | **Q11** | **Q12** | **Q13** |
| --- | --- | --- | --- | --- | --- | --- | --- | --- | --- | --- | --- | --- | --- |
| Barbalho et al. (2019) | Y | U | Y | Y | N | U | Y | Y | Y | Y | U | Y | U |
| Ogawa et al. (2021) | U | U | U | N | N | U | Y | Y | Y | Y | Y | Y | Y |

For the two randomized studies, the JBI trial checklist was applied to the randomized study design. The participant-characteristic analyses relevant to this scoping review were secondary/exploratory analyses and were not interpreted as formal treatment-effect modification unless an interaction between the characteristic and BFR exposure had been tested.

| **Item** | **JBI item** |
| --- | --- |
| **Q1** | Was true randomization used for assignment of participants to treatment groups? |
| **Q2** | Was allocation to treatment groups concealed? |
| **Q3** | Were treatment groups similar at baseline? |
| **Q4** | Were participants blind to treatment assignment? |
| **Q5** | Were those delivering treatment blind to treatment assignment? |
| **Q6** | Were outcome assessors blind to treatment assignment? |
| **Q7** | Were treatment groups treated identically other than the intervention of interest? |
| **Q8** | Was follow-up complete and, if not, were differences between groups in follow-up adequately described and analyzed? |
| **Q9** | Were participants analyzed in the groups to which they were randomized? |
| **Q10** | Were outcomes measured in the same way for treatment groups? |
| **Q11** | Were outcomes measured in a reliable way? |
| **Q12** | Was appropriate statistical analysis used? |
| **Q13** | Was the trial design appropriate, and were any deviations from a standard parallel-group RCT accounted for in conduct and analysis? |
